# Restoration of IFNγR Subunit Assembly, IFNγ Signaling and Parasite Clearance in *Leishmania donovani* Infected Macrophages: Role of Membrane Cholesterol

**DOI:** 10.1371/journal.ppat.1002229

**Published:** 2011-09-08

**Authors:** Subha Sen, Koushik Roy, Sandip Mukherjee, Rupkatha Mukhopadhyay, Syamal Roy

**Affiliations:** Division of Infectious Diseases and Immunology, Indian Institute of Chemical Biology, Council of Scientific and Industrial Research, Kolkata, India; National Institutes of Health, United States of America

## Abstract

Despite the presence of significant levels of systemic Interferon gamma (IFNγ), the host protective cytokine, Kala-azar patients display high parasite load with downregulated IFNγ signaling in *Leishmania donovani* (LD) infected macrophages (LD-MØs); the cause of such aberrant phenomenon is unknown. Here we reveal for the first time the mechanistic basis of impaired IFNγ signaling in parasitized murine macrophages. Our study clearly shows that in LD-MØs IFNγ receptor (IFNγR) expression and their ligand-affinity remained unaltered. The intracellular parasites did not pose any generalized defect in LD-MØs as IL-10 mediated signal transducer and activator of transcription 3 (STAT3) phosphorylation remained unaltered with respect to normal. Previously, we showed that LD-MØs are more fluid than normal MØs due to quenching of membrane cholesterol. The decreased rigidity in LD-MØs was not due to parasite derived lipophosphoglycan (LPG) because purified LPG failed to alter fluidity in normal MØs. IFNγR subunit 1 (IFNγR1) and subunit 2 (IFNγR2) colocalize in raft upon IFNγ stimulation of normal MØs, but this was absent in LD-MØs. Oddly enough, such association of IFNγR1 and IFNγR2 could be restored upon liposomal delivery of cholesterol as evident from the fluorescence resonance energy transfer (FRET) experiment and co-immunoprecipitation studies. Furthermore, liposomal cholesterol treatment together with IFNγ allowed reassociation of signaling assembly (phospho-JAK1, JAK2 and STAT1) in LD-MØs, appropriate signaling, and subsequent parasite killing. This effect was cholesterol specific because cholesterol analogue 4-cholestene-3-one failed to restore the response. The presence of cholesterol binding motifs [(L/V)-X_1–5_-Y-X_1–5_-(R/K)] in the transmembrane domain of IFNγR1 was also noted. The interaction of peptides representing this motif of IFNγR1 was studied with cholesterol-liposome and analogue-liposome with difference of two orders of magnitude in respective affinity (K_D_: 4.27×10^−9^ M versus 2.69×10^−7^ M). These observations reinforce the importance of cholesterol in the regulation of function of IFNγR1 proteins. This study clearly demonstrates that during its intracellular life-cycle LD perturbs IFNγR1 and IFNγR2 assembly and subsequent ligand driven signaling by quenching MØ membrane cholesterol.

## Author Summary

The disease Visceral Leishmaniasis or Kala-azar is extending its base in the Indian subcontinent and elsewhere. The emergence of drug resistant cases is aggravating the problem further. The kala-azar patients do not respond to the host-protective cytokine IFNγ at the active stage of the disease, the cause of which is unknown. This research is designed to understand how cell surface receptors for IFNγ respond under parasitized condition. Our results clearly showed that the *Leishmania* parasites during their intracellular life-cycle make the host cell membrane fluid by quenching cholesterol from the membrane, which renders the IFNγR nonfunctional despite their physical presence on the cell surface. Upon supplementation of cholesterol in infected MØs, the infected cells regain responsiveness to IFNγ coupled with intracellular parasite killing. Thus supplementation of cholesterol together with IFNγ may be a new approach to treat drug unresponsive Kala-azar cases.

## Introduction

Visceral Leishmaniasis (VL), a potentially fatal visceralizing disease, afflicts millions of people worldwide and is caused by infection with *Leishmania donovani* (LD), an obligate-intracellular trypanosomatid protozoan. During the past decades, a large body of evidences supported the notion that the cytokine interferon gamma (IFNγ) plays a decisive role in anti-leishmanial defense [1], [2]. A primary defect that may lead to pathogenesis in VL is the failure to activate parasitized macrophages (MØs) to eliminate LD in response to IFNγ [3]. Intriguingly, the presence of elevated levels of serum IFNγ in human VL [4]–[8] and high expression of IFNγ mRNA in lymphoid organs [9] do not reconcile with large parasite burden observed at the active stage of the disease. The remarkable predominance of the Th1 cytokine IFNγ along with impaired MØ effector function indicates a MØ specific desensitization to the available IFNγ stimulus. This was evident from several *in vitro* studies [3], [10], [11] showing gross inhibition of the IFNγ signaling pathways in the LD infected MØs (LD-MØs), but the exact mechanism that triggers the inhibition remained unknown till date.

IFNγ binds to specific cell surface receptor IFNγR which consists of two heterodimeric subunits, IFNγR1 (α, ligand binding subunit) and IFNγR2 (β, signal-transducing subunit). Signal transduction of IFNγ is initiated by its binding to IFNγR1 and subsequent receptor subunit multimerization [12]. IFNγR1 colocalizes partly with the ganglioside GM1, a classical marker of specialized cholesterol-rich membrane microdomains termed lipid-rafts [13]. Subsequent evidences disclosed that membrane lipid-rafts are intimately involved in the process of IFNγ mediated signal transduction. Remarkably, irrespective of various cell types used in different reports, disruption of the plasma membrane rafts by cholesterol depletion using methyl-β-cyclodextrin (mBCD) or cholesterol sequestration with filipin reversibly affected not only the generation of the IFNγ inducible tyrosine phosphorylation of signal transducer and activator of transcription 1 (STAT1) but also productive transcriptional signaling [14]–[16]. This is congruent with the prevalent hypothesis of lipid raft involvement in IFNγ mediated signaling [17]. Membrane proteins such as G protein coupled receptors, transporters, or ion channels have been shown to be localized or enriched in lipid rafts. The structure- activity analysis shows that many of the cholesterol effects are due to specific sterol-protein interactions as shown in case of a number of membrane bound receptors such as those for cholecystokinin (type B), oxytocin and nicotinic acetylcholine (nACh) [18]. Refined structure of nACh receptor (nAChr) has been shown to have internal sites capable of containing cholesterol, whose occupation bolsters the protein structure [19]. Both oxytocin and serotonin_1A_ receptors contain the strict cholesterol consensus motif (CCC), and in both the cases cholesterol incorporation lead to dramatic increase in agonist affinity [20], [21].

Previous studies from our group have shown that during their intracellular life-cycle LD parasites disrupt the membrane rafts of MØs by quenching cholesterol from the membrane. This was associated with defective antigen presenting function, which could be corrected by liposomal delivery cholesterol [22], [23]. We also showed that systemic administration of cholesterol through liposomal delivery in LD infected hamsters has strong therapeutic efficacy [23], in agreement with the clinical practice of using cholesterol either directly [24] or as an adjunct to treatment [25]. It may be recalled that Kala-azar patients show progressive decrease in serum cholesterol as a function of splenic parasite load [26]. Therefore we investigated the potential involvement of LD mediated manipulation of MØ membrane cholesterol in IFNγ signaling modulation.

Herein we report for the first time that the earliest activation induced signaling event of IFNγ receptor oligomerization is inhibited due to quenching of the MØ membrane cholesterol by the LD parasites. Restoration of the MØ membrane cholesterol by exogenous delivery not only reinstates the receptor subunit interaction but also restores subsequent downstream signaling as well as the IFNγ mediated leishmanicidal property.

## Results

### Defective IFNγR subunit multimerization in LD infected MØs

Previous reports revealed that *Leishmania* spp. infected MØs are refractory to exogenous IFNγ mediated clearance of intracellular parasites due to defective IFNγ signaling [3, 11, 27, and 28]. In this study, the molecular mechanism/s triggering the impairment of IFNγ signaling in LD-MØs have been investigated.

To investigate the mechanistic basis of defective IFNγ signaling in LD-MØs, BALB/c derived peritoneal MØs (PEC) and homologous cell-line of RAW 264.7 were used. While performing the preliminary experiments (Figure S1-S3), RAW 264.7 cells yielded results comparable to the native system of PEC; therefore all the subsequent experiments were performed using these cells unless otherwise mentioned.

Of note, JAK2 phosphorylation, which is the proximal most activation event down-stream to IFNγR1 engagement [12], [29]–[32] was uncoupled (Figure S3A), from the intact IFNγ-IFNγR1 interaction in LD-MØs (Figure S2B). It has been reported that ligand induced receptor multimerization is the crucial and sufficient trigger for the activation of most of the cytokine receptors [33]–[36], including IFNγ receptors [37]–[41]. Thus, we hypothesized that abrogation of earliest signal of JAK2 phosphorylation by IFNγR1 engagement in LD-MØs most likely involve the impairment of immediate upstream event of IFNγR subunit multimerization.

**Figure 1:**
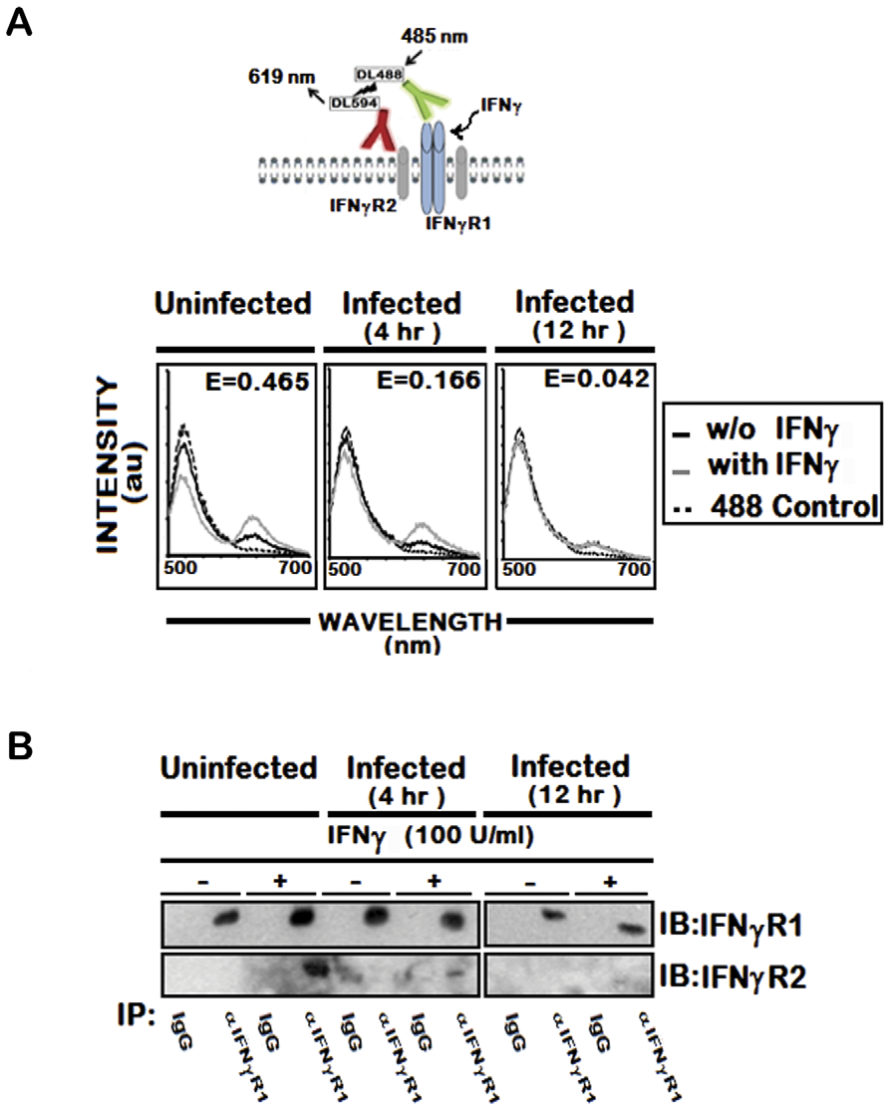
IFNγ induced IFNγR subunit association is impaired by LD infection. **A,** Fluorescence spectrometric analysis of IFNγR1-IFNγR2 FRET in uninfected and infected RAW 264.7 cells. Cells were treated with/without 100 U/ml of rIFNγ for 3 minutes, washed with ice cold PBS, transferred to ice and then fixed immediately. Cell-surface IFNγR1 and IFNγR2 were then stained with respective mAb linked directly to fluorophores, Dylight488 (DL488) for anti-IFNγR1 and Dylight594 (DL594) for anti-IFNγR2. The fluorescence emission spectra in response to 485 nm excitation before (solid black) and after (solid grey) IFNγ treatment are shown with hashed black line representing the single stained Dylight488 control. ‘E’ in the figures represents the FRET efficiency of IFNγ treated samples, calculated as described in *Materials and Methods*. **B**, Immunoblot Analysis of IFNγR1 and IFNγR2 in immunoprecipitates of anti-IFNγR1. IFNγR1was immunoprecipitated with either specific or control immunoglobulin from uninfected and infected MØs stimulated with/without 100 U/ml of rIFNγ for 3 minutes as described in *Materials and Methods*. Copurifying IFNγR2 was detected by immunoblotting with anti-IFNγR2 (lower gel).

To address this possibility, we utilized the fluorescence resonance energy transfer (FRET) method, a powerful spectrofluorometric technique that has been used to determine molecular interactions with higher resolution [42]. We assessed FRET between cell surface IFNγR1 and IFNγR2 molecules that were fluorescently labeled with receptor specific antibodies tagged with DyLight488 or DyLight594 respectively. As shown in Figure 1A (panel a), the emission spectrum excited with 485 nm light showed reciprocal changes in donor emission peak (517 nm) versus acceptor emission (620 nm), indicating that IFNγ treatment promoted energy transfer between IFNγ-R1 and -R2 in normal cells. In marked contrast, stimulation of LD infected cells with IFNγ could induce minimal or no FRET signal between IFNγR1 and IFNγR2 (Figure 1A, panel b and c). The FRET efficiency was calculated in each of these conditions. As this was found to be low in infected cells (E = 0.166 in 4 hr and E = 0.042 in 12 hr LD infection) compared to that (E = 0.465) of normal IFNγ stimulated condition, LD infection must have significantly inhibited ligand induced receptor association. Thus the results indicate prominent reduction in the ligand induced IFNγR1-IFNγR2 FRET efficiency, which is reduced by 64.6 % at 4 hr and 90.9 % at 12 hr post LD infection than the normal condition.

To confirm the observation, we coimmunoprecipitated IFNγR1 and IFNγR2 from ligand stimulated or unstimulated normal and infected MØs. The results (Figure 1B) indicate that IFNγ induced copurification of the IFNγR2 subunits with IFNγR1 in normal condition of the MØs. Conversely, infection for 4 hr markedly reduced the extent of IFNγR2 coprecipitation with IFNγR1 even in IFNγ stimulated condition (Figure 1B), while the level of coprecipitated IFNγR2 went below detection level with increasing time of infection. Taken together, the results in this section imply that LD infection inhibits IFNγ induced receptor oligomerization in MØs.

### Ligand induced IFNγR subunit partitioning in detergent resistant membrane fraction is lost in LD infected MØs

Association with lipid-raft membrane domains is known to regulate the oligomerization of various cell surface receptors [43]–[45]. It is also accepted that localization of the IFNγR complex within intact lipid raft microdomains governs their efficient signal transduction capability [14]–[16]. The aforementioned results suggested that LD infection inhibits IFNγ induced receptor oligomerization in MØs, despite having unaltered initial ligand-receptor association. We therefore speculated that differential membrane partitioning behavior of the subunits might affect the IFNγ-R1 and -R2 interaction. It was therefore necessary to examine whether LD infection of the host cells delocalizes the IFNγR chains from the lipid raft microdomains that in consequence might hinder efficient oligomerization of the receptor subunits upon IFNγ mediated stimulation.

**Figure 2:**
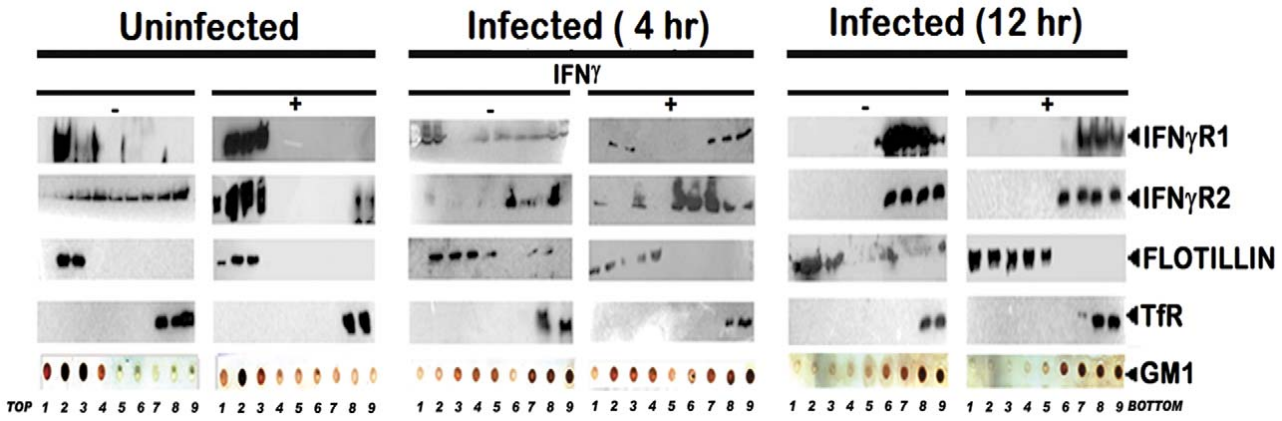
Disruption of lipid raft microdomains suppresses IFNγR subunit assembly in LD infected MØs. Uninfected and LD infected MØs left untreated or treated with 100 U/ml of rIFNγ (3 minutes, 37°C) were immediately washed with ice-cold PBS and crude membranes were prepared which were then extracted with 1% Triton X-100 and subjected to optiprep density gradient centrifugation to isolate lipid rafts. Proteins from equal volume of aliquots of the collected fractions were separated by SDS-PAGE and analyzed by western blotting using specific Ab against IFNγR1, IFNγR2, Flotillin 1 and Transferrin receptor. To analyze the distribution of GM1, 2 µl of each fraction was dot blotted onto a nitrocellulose membrane and detected using CTxB-HRP. ‘TfR’ denotes transferrin receptor for this and the subsequent experiments.

To study the abrogation of IFNγ induced IFNγR subunits partitioning, if any, under parasitized conditions, MØ membrane extracts with 1% Triton X-100 were subjected to optiprep density gradient separation and examined by immunoblotting to analyze the distribution of IFNγR subunit proteins (Figure 2). Cholesterol-rich lipid raft domains were characterized by the enrichment of known raft marker protein flotillin1, while the fractions containing the non-raft portions were identified with the surrogate marker of transferrin receptor [46], [22]. The success of the biochemical isolation process was confirmed by determining the lipid raft constituent glycosphingolipid GM1 in each fraction by dot blot. Careful analysis of the fractionation study revealed that the constitutive association of IFNγR1 with the detergent resistant lipid raft fractions was discernibly disoriented at steady-state of the early infected MØs where appreciable amount of IFNγR1 was present in the detergent soluble fractions. Whereas, with increasing severity of the LD infection, the IFNγR1 protein was nearly completely delocalized from the lipid raft fractions and were recovered from the detergent soluble higher density fractions (Figure 2). On the contrary, the localization pattern of the IFNγR2 subunit showed a more or less diffused distribution throughout the density gradients of the steady state of the normal MØs. Upon IFNγ mediated activation, the IFNγR2 subunit is recruited to the lipid raft fractions almost to entirety which is accompanied by robust receptor subunit interaction, as indicated in the above mentioned energy transfer and coIP experiments (Figure 1). Intriguingly, the IFNγR2 subunit distribution pattern was markedly altered from the very early hours of infection through the late hours, when bulk of the proteins were recovered from the non-raft fractions, denoting complete detergent solubilization. Nevertheless, at early hours of LD infection stimulation of the cells with IFNγ could elicit IFNγR2 redistribution albeit to a lesser extent and this partial IFNγR2 recruitment to the raft domains was reflected in concomitant decline in the efficiency of receptor subunit interaction and signaling potency. Notably, at late hours of LD infection, despite having simultaneous presence of both IFNγR1 and IFNγR2 within the non-raft fractions, the IFNγ mediated stimulation of the MØs failed to induce any detectable subunit interaction as evidenced by the absence of FRET or coIP positivity at 12 hr postinfection.

**Figure 3:**
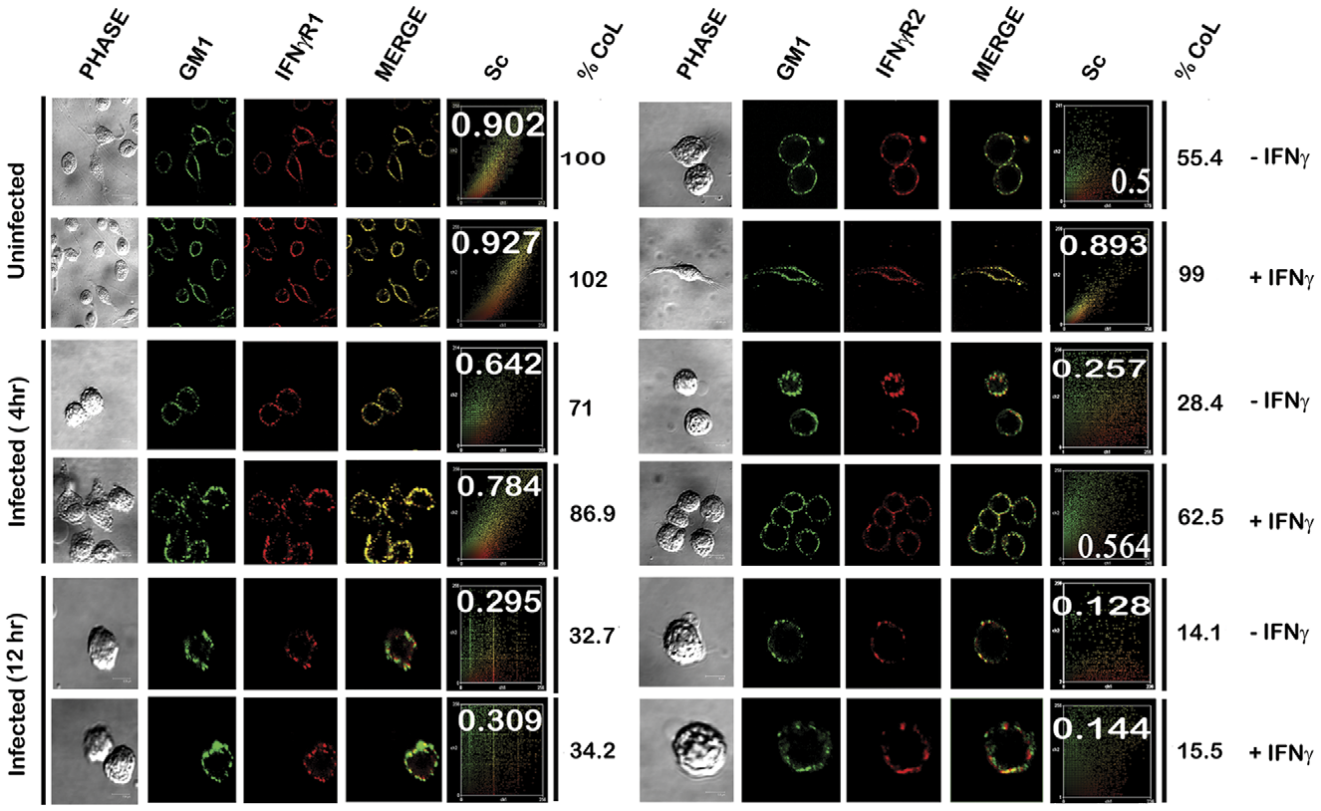
Confocal colocalization analysis of the IFNγR subunits in uninfected and LD infected macrophages. RAW 264.7 left untreated or treated with 100 U/ml of rIFNγ were stained with FITC-CTxB followed by either anti-IFNγR1 or anti-IFNγR2 followed by Fc specific secondary and streptavidin-PE. The associated scatterplot represents the extent of colocalization of each experimental set, with inset showing the calculated Pearson's correlation coefficient (PCC) values. The percentage of colocalization was estimated as a function of the basal colocalization of IFNγR1 protein with the GM1 molecule in minimal invasive condition (without any manipulation of the steady-state condition of the naïve MØs). The values under the column of % CoL denote the respective percent colocalization of individual protein with GM1 in the respective experimental conditions. ‘Sc’: Scatterplot.

The results were further validated *in situ* by confocal colocalization study, which recapitulated the observations of the floatation studies for the receptor distribution patterns. Analysis of the colocalization pattern of the individual subunits of the IFNγR assembly with the GM1 rich microdomains was quantified and expressed in terms of the Pearson's correlation coefficient (PCC) values. The PCC values obtained by colocalization analysis of constitutively raft associated IFNγR1 proteins with GM1 at the steady state of the naïve MØs were considered as basal, and each experimental condition assessed thereafter was represented as the function of this optimal (100%) colocalization efficiency. The results in Figure 3, demonstrate that the percentage colocalization of IFNγR1 with GM1 molecules upon IFNγ mediated activation of the normal MØs remain unchanged (102%), signifying preassociation of the IFNγR1 protein with the GM1 enriched domain. The results also reveal that, there is a considerable decrease in the percentage of colocalized IFNγR1 with GM1 in the macrophage membrane at 4 hour post LD infection (71%) than the basal condition. However, IFNγ stimulation could elicit a minor increase in the colocalization pattern (86.9%) sufficient to elicit a partial activation of signaling cascade. With increasing time of infection at 12 hr post infection, we observed a sharp decline in the percentage of the colocalized IFNγR1 (32.7%) along with absolute failure of IFNγ to induce any detectable increase in the colocalization pattern at this condition (34.2%).

Comparison of the percentage colocalization of the IFNγR2 with GM1 at the steady-state of the MØs showed a steep decline both in the early as well as the late hours of LD infected MØs than the uninfected control. In agreement with the floatation studies, IFNγ activation could elicit a partial increase in the colocalization efficiency at the early hours of LD infection (62.5%), though absence of the same at late hours of infection denotes a conspicuous block in the IFNγ triggered translocational behavior of IFNγR2.

### Impaired IFNγ signaling complex formation under parasitized condition

**Figure 4:**
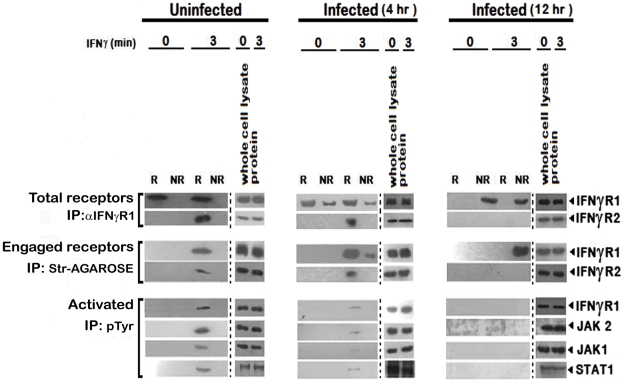
Repression of IFNγR subunit assembly in disrupted lipid raft microdomains in LD infected MØs abrogates productive IFNγ signaling. Analysis of accumulation of the IFNγ induced signaling complex in lipid rafts of RAW 264.7 cells. MØs were left uninfected or infected with LD and stimulated with/without 0.5 ng/ml biotinylated IFNγ for 3 min after the indicated time points of infection. Triton X-100 soluble non-raft (NR) and insoluble lipid raft (R) fractions were isolated as described in figure 2. The fractions 2–4 were pooled to yield a total raft (R) compartment and fractions 7–9 were pooled to obtain the non-raft (NR) compartment. Engaged receptors and their signaling complexes were immunoprecipitated using Streptavidin-agarose beads. For comparison, the total amount of receptor was immunoprecipitated by adding anti-IFNγR1 Ab to the soluble non-raft and raft fractions. Immunoprecipitates and corresponding total cell lysates were subjected to SDS-PAGE and immunoblotted using specific Ab against IFNγR1 and IFNγR2. Activated forms of the signaling subunits were determined by dissociating the engaged signaling complexes pulled down by Streptavidin-agarose beads, by boiling in 1% SDS, followed by immunoprecipitation using an anti-pTyr Ab. Activated and phosphorylated proteins were analyzed by western blotting using Ab against IFNγR1, JAK2, JAK1 and STAT1. Equal loading was verified by immunoblotting of respective whole proteins in whole cells lysates of each experimental set. Str: Streptavidin.

To investigate further, we stimulated the uninfected and LD infected MØs with biotinylated IFNγ and isolated the lipid rafts. Total receptors and engaged receptors were immunoprecipitated using anti-IFNγR1 and streptavidin-agarose respectively. The activated forms of the signaling complex were determined by resolubilization of the immunoprecipitates of streptavidin-agarose, reprecipitation by p-Tyr antibody and immunoblotting with antibodies against distinct components of the IFNγ signaling complex namely IFNγR1, JAK1, JAK2 and STAT1. As depicted in Figure 4, endogenous IFNγR1 distribution was less affected during early (4^th^) hours of infection, but with increasing severity of infection it was completely delocalized from the raft fractions. Most striking of these results is that, though we could recover some ligand engaged IFNγR1 from non-raft portions at 4 hr post infection, IFNγR2 was only detectibly coimmunoprecipitated from the raft fractions. Another significant finding of this experiment was that though endogenous IFNγR1 and engaged IFNγR1 could be immunoprecipitated from both raft and non-raft fractions after 3 minutes of IFNγ stimulation from 4 hr LD infected macrophages, the phosphorylated (activated) IFNγR1 was only detected in the immunecomplexes from the raft fractions along with other activated signaling intermediates. It is worthy of note that at late hours of infection, though engaged IFNγR1 (Figure 4) and IFNγR2 (Figure 2) were simultaneously present in the non-raft fractions, they could not generate any productive interaction as evidenced by the complete absence of IFNγ triggered CoIP and/or phosphorylation signal. These results denote critical importance of cholesterol rich microenvironment for proper IFNγR functioning.

### Cholesterol dependent modulation of IFNγ mediated receptor subunit interaction in LD infected MØs involving differential raft partitioning of IFNγR subunits

**Figure 5:**
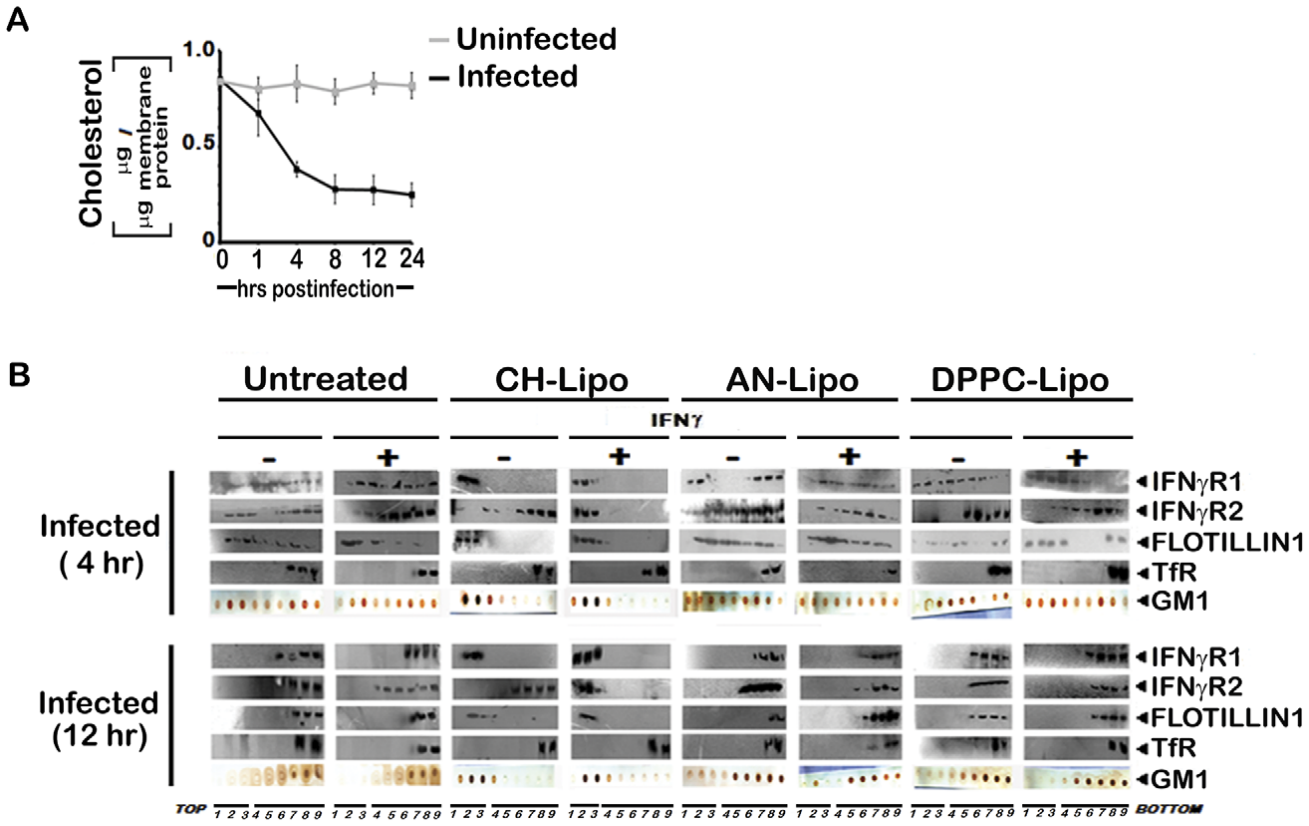
Membrane cholesterol influences the submembrane distribution of IFNγR subunits as well as IFNγ induced receptor subunit interaction and signaling efficacy in LD infected MØs. **A**, Analysis of membrane cholesterol of uninfected and infected MØs. Cholesterol content in isolated membrane fractions was measured as indicated in *Materials and Methods*. The data represents mean ± SD of triplicate analyses. **B**, Restoration of IFNγ inducible IFNγR subunit translocation in membrane rafts in cholesterol repleted LD infected MØs. LD infected MØs left untreated or treated with Cholesterol-liposome (CH-Lipo) or 4-cholestene-3-one-liposome (AN-Lipo) or DPPC-liposome (DPPC-Lipo), were stimulated with 100 U/ml of rIFNγ (3 minutes, 37°C) at indicated timepoints postinfection, immediately washed with ice-cold PBS and subjected to membrane harvestation. Membrane preparation and gradient fractions were done as described in figure 2. Equal volume of fractions from each optiprep gradient were analyzed by western blotting by using antibodies to IFNγR1, IFNγR2, Flotillin 1 and Transferrin receptor (TfR) and GM1 dot blots using CTxB-HRP.

**Figure 6:**
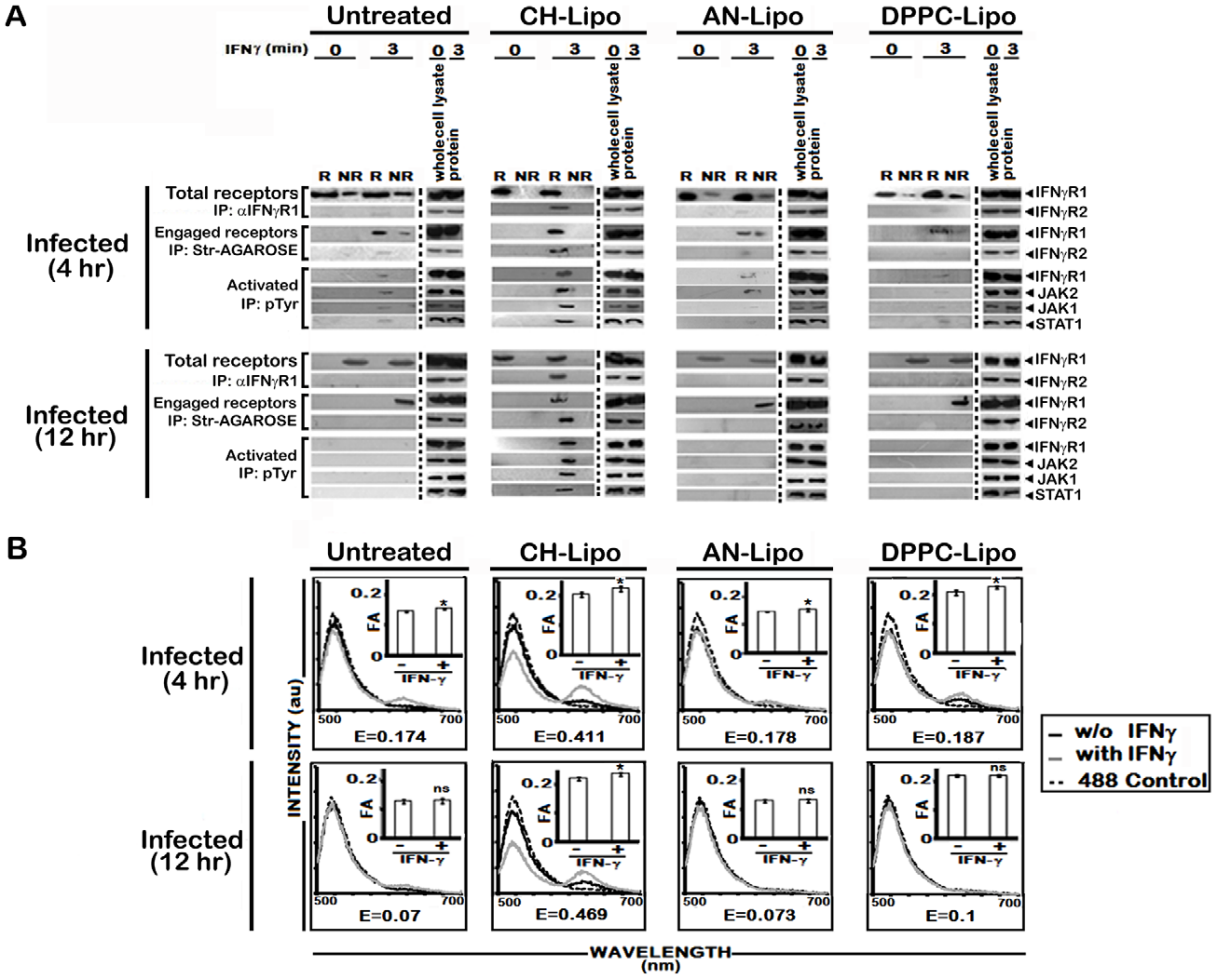
Analysis of raft association of steady-state and activated IFNγR signaling complex in LD infected and differentially treated MØs. **A**, Immunoblot analysis of immunoprecipitates of isolated raft (R) and non-raft (NR) fractions from LD infected MØs left untreated or treated with either Cholesterol-liposome (CH-Lipo) or 4-cholestene-3-one-liposome (AN-Lipo) or DPPC-liposome (DPPC-Lipo), and stimulated with 0.5 ng/ml of biotinylated IFNγ (3 minutes, 37°C) at indicated timepoints postinfection. Either anti-IFNγR1 or streptavidin-agarose beads were used for precipitating immunocomplexes associated with total endogenous cell surface IFNγR1 or IFNγ engaged IFNγR1 as described in details in *Materials and Methods.* Activated forms of the signaling subunits were determined by resolubilizing the engaged signaling complexes followed by immunoprecipitation using an anti-pTyr Ab. Activated and phosphorylated proteins were analyzed by western blotting using Ab against IFNγR1, JAK2, JAK1, and STAT1. Immunoblotting of respective whole proteins in whole cells lysates of each experimental set verified equal loading. **B**, Analysis of ligand inducible IFNγR subunit interaction in LD infected and differentially treated MØs. Fluorescence spectrometric assay of IFNγR1-IFNγR2 FRET in infected RAW 264.7 cells left untreated or treated with Cholesterol-liposome (CH-Lipo) or 4-cholestene-3-one-liposome (AN-Lipo) or DPPC-liposome (DPPC-Lipo). Cells were treated with/without 100 U/ml of rIFNγ for 3 minutes at 37°C and washed and transferred to ice then fixed immediately. FRET analyses were done exactly as described in figure legend 1A. *Insets* Measurements of fluorescence anisotropy (FA) value similarly treated cells as in figure 6B. ‘E’ represents the FRET efficiency of IFNγ treated samples, calculated as described in *Materials and Methods*.

The involvement of rafts in early IFNγ signaling events such as induction of STAT1 tyrosine phosphorylation has previously been studied using methyl-ß-cyclodextrin (mBCD) as well as filipin and nystatin [14]–[16], all of which perturb the raft integrity via cholesterol depletion or sequestration [47]. Extending and verifying our original observation of decreased MØ membrane cholesterol in LD infected *in vivo* model [23], our *in vitro* system of infected RAW 264.7 cells also showed reduction in membrane cholesterol as function of time (Figure 5A), the kinetics of which exactly coincided with the time points of waning IFNγ signaling. To clearly establish the role of cholesterol in IFNγ mediated receptor assembly, we replenished the LD-MØs with exogenous cholesterol in liposomal form (CH-Liposome). Figure 5B shows that cholesterol supplementation induced relocalization of IFNγR1 within the raft domains regardless of the time points of investigation after LD infection. Interestingly, in cholesterol replete MØs, the engaged receptors were entirely recovered from the raft fractions (Figure 6A). This cholesterol induced IFNγR1 relocalization was prominently associated with a marked increase in IFNγR2 interaction, which was readily detected from the immunecomplexes isolated by total IFNγR1 and/or engaged IFNγR1 in the IFNγ stimulated condition (Figure 6A). The restored pattern of IFNγR subunit multimerization was more conclusively verified in FRET experiments, where cholesterol supplementation reinstated the IFNγ inducible receptor subunit interactions (Figure 6B).

To establish unambiguously the specific role of cholesterol in influencing the earliest step of IFNγR signaling in reconstituted membrane rafts of LD-MØs, we treated the infected MØs with liposomes prepared with oxidized cholesterol analogue of 4-cholestene-3-one (AN-Liposome). The results of coimmunoprecipitation (Figure 6A) and FRET (Figure 6B) analyses between IFNγR subunits in AN-Liposome treated infected MØs showed that treatment with AN-liposome failed to restore IFNγR translocations as also the subunit interactions by IFNγ stimulation.

In order to investigate whether the enhanced IFNγR oligomerization in cholesterol-replete cells directly involves action of cholesterol rather than its indirect effect via increased bulk membrane rigidity, we treated LD-MØs with DPPC, a phospholipid previously shown to induce increased membrane rigidity in model membranes [48]. The data in Figure 5B, 6A reveal that in contrast to cholesterol supplemented cells, the DPPC treated cells, despite having comparable bulk membrane rigidity as indicated by the FA values (Figure 6B, insets), failed to induce IFNγ receptor interaction indicating specific requirement of cholesterol in IFNγR subunit assembly.

**Figure 7:**
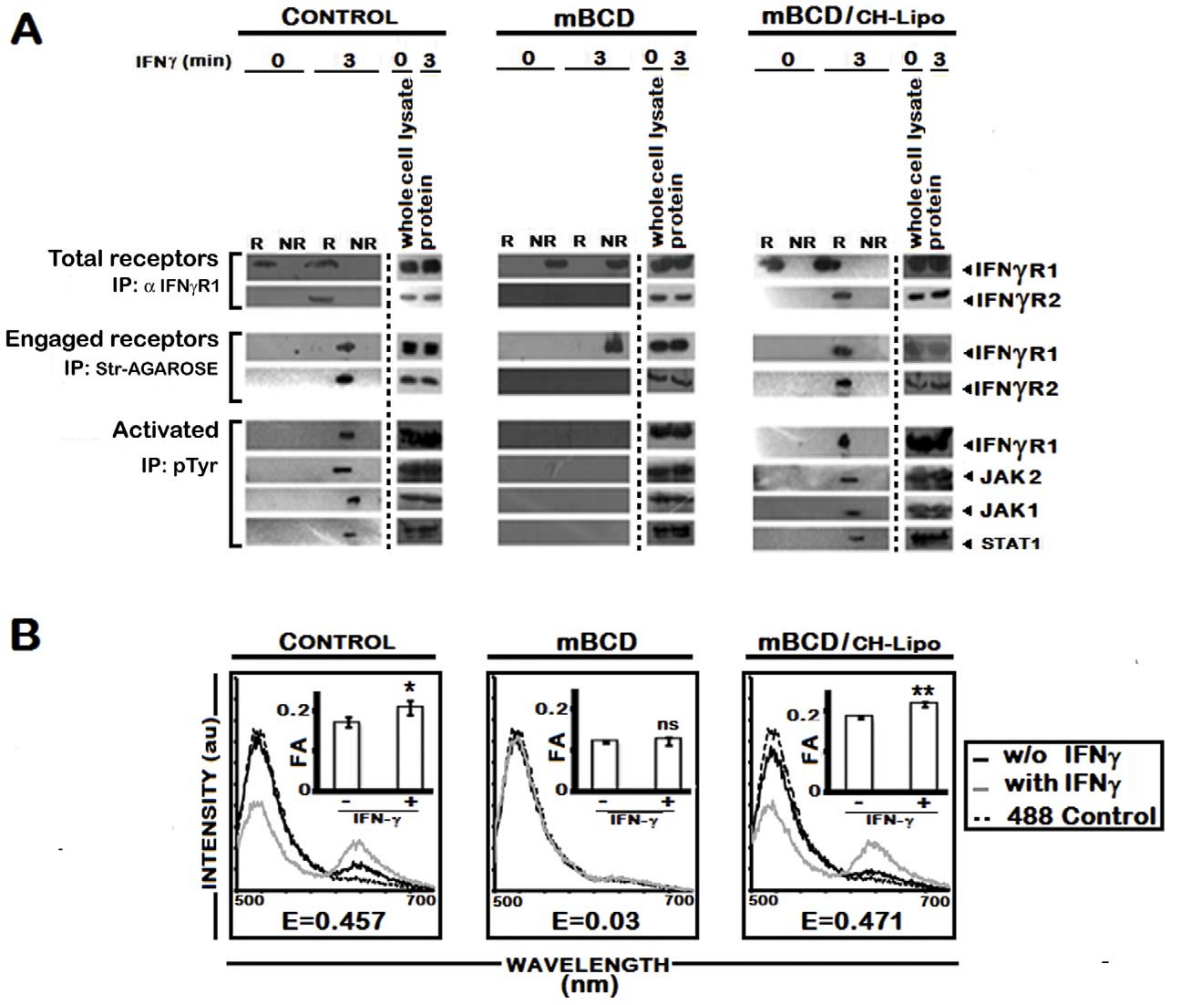
Effect of pharmacological depletion of cholesterol from MØs on IFNγ signaling and IFNγR subunit interaction. **A**, Analysis of raft association of steady-state and activated IFNγ signaling complex in mBCD treated and cholesterol repleted MØs. Confluent RAW 264.7 cells were subjected or not (*Control*) to cholesterol depletion using 5 mM mBCD for 30 minutes at 37°C, and to cholesterol reloading using Cholesterol-liposomes (CH-Lipo) (*repletion*). Following these treatments, cells were stimulated or not with 0.5 ng/ml of biotinylated IFNγ for 3 minutes, 37°C, before harvesting of cells. Total endogenous cell surface IFNγR1 and ligand engaged IFNγR1 were precipitated from pooled raft (R) (fractions 2–4) and non-raft (NR) compartments (fractions 7–9) by anti-IFNγR1 or streptavidin-agarose beads as described in *Materials and Methods*. The immune complexes were analyzed by Western blot using the appropriate antibodies against IFNγR1 and IFNγR2. Equal loading was verified by respective whole protein immunoblotting from corresponding whole cell lysates. A sequential immunoprecipitation with pTyr antibody experiment from the resolubilized immunecomplexes confirmed the activation status of the signaling subunits when blotted with antibodies against IFNγR1, JAK2, JAK1 and STAT1. **B**, FRET analysis of ligand induced IFNγR subunit interaction in mBCD treated and cholesterol repleted MØs. Fluorescence spectrometric assay of IFNγR1-IFNγR2 FRET in RAW 264.7 cells that were either left untreated (*Control*) or subjected to cholesterol depletion or cholesterol reloading of the depleted cells using Cholesterol-liposomes (CH-Lipo) (*repletion*). Cells were treated with/without 100 U/ml of rIFNγ for 3 minutes, 37°C and washed with ice cold PBS and transferred to ice then fixed immediately. FRET analyses were done exactly as described in figure legend 1A. *Insets* Measurement of fluorescence anisotropy (FA) value of corresponding experimental RAW 264.7 cells treated with/without 100 U/ml of rIFNγ. Data represents mean ± SD. ‘E’ represents the FRET efficiency of IFNγ treated samples, calculated as described in *Materials and Methods*.

To corroborate the finding of cholesterol specific IFNγR subunit interaction, RAW 264.7 cells were treated with mBCD, which disrupts lipid rafts by specific depletion of cholesterol [49]. As shown in Figure 7A, stimulation of mBCD treated cells with IFNγ failed to induce association of IFNγR2 subunit with engaged IFNγR1, which was found to be localized in the non-raft membrane fractions. Comparable to the LD infected macrophages, mBCD treatment completely abolished the activation of IFNγ signaling components while cholesterol replenishment of these cells restored the subunit assembly (Figure 7) and activation pattern of IFNγ induction (Figure 7A). In additional control experiments, conditions of mBCD treatments in RAW 264.7 cells were first determined to ensure unaffected cell viability and unaltered expression of surface IFNγR and components of STAT1 activation axis (data not shown).

### LPG plays no role in delocalization of IFNγR from raft

**Figure 8:**
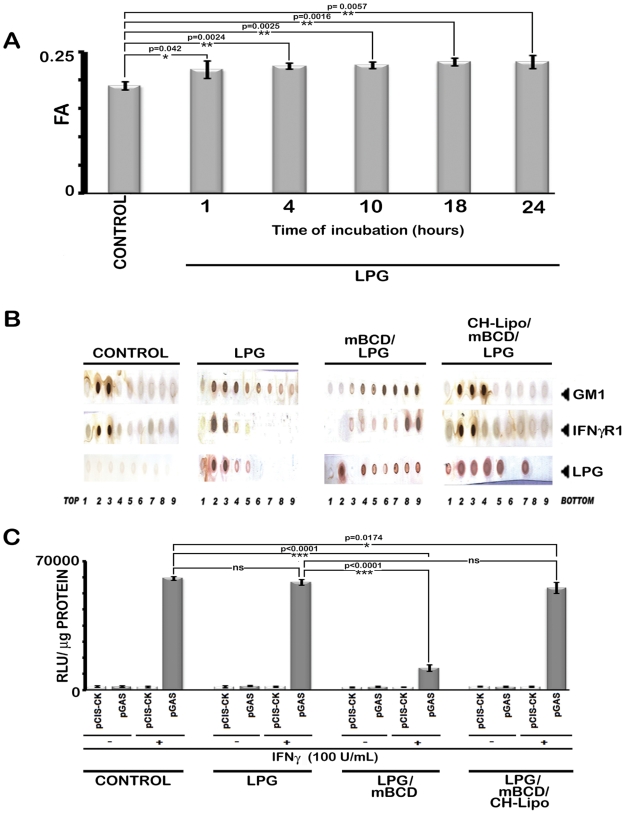
Analysis of LPG-MØ interactions. **A,** Measurements of FA value of RAW 264.7 cells left untreated or treated with 10 µg/ml of purified LPG for varying time periods. **B**, RAW 264.7 cells left untreated or treated with 10 µg/ml of purified LPG for 18 hrs (time equivalent of total parasite-MØ interaction of 6 hrs of initial attachment and 12 hours of infection establishment) were subjected to membrane harvestation. Gradient fractions were purified as described in figure 2. Equal volume of fractions from each optiprep gradient were analyzed by dot blot for the presence of GM1, IFNγR1, LPG by using the appropriate antibodies and CTxB-HRP. **C,** IFNγ induced bioactivity in LPG treated MØs was determined by reporter assay. RAW 264.7 cells were transfected with pCIS-CK negative control vector or IFNγ driven pGAS reporter plasmid. 12 hrs after transfection, cells were either left untreated or treated with mBCD (5 mM, 30 min, 37°C) and /or treated with 10 µg/ml of LPG for 18 hours. Treated cells were then replenished or not with CH-Lipo. Subsequently treated cells were stimulated with or without 100 U/ml of rIFNγ eight hours before final harvesting for luciferase activity determination in cell lysates. The data represents the relative luciferase activity normalized to per microgram of protein content. Data are representative of three independent experiments.

LPG is a GPI anchored molecule secreted/shed by the parasites after internalization in MØs by phagocytosis [50], [51]. It is known to increase the total membrane rigidity. Consistent with previous reports [52]–[54], LPG induced significantly higher bulk membrane rigidity upon incubation with MØs (Figure 8A) and was incorporated substantially within GM1 enriched membrane domains. However, it could not induce IFNγR1 delocalization from the more buoyant lipid raft fractions as indicated by dot blot analyses of individual membrane fractions of optiprep gradient (Figure 8B). This intactness of IFNγR1 localization in the LPG incubated MØs was also reflected in undiminished IFNγ functionality in these cells as assessed by the gamma activated sequence driven luciferase (GAS-Luc) reporter system (Figure 8C), a finding supported previously by Ray *et al.* [11]. Notably, pharmacological depletion of membrane cholesterol from the LPG-incubated MØs completely disoriented the IFNγR1 localization pattern and disrupted the membrane rafts as indicated by the even distribution of GM1 molecules along the isolated density gradients of the cell membrane (Figure 8B). Importantly, repletion of cholesterol in the system of LPG-incubated mBCD-treated MØs restored both the localization and functionality pattern of IFNγR1 (Figure 8B, 8C).

**Figure 9:**
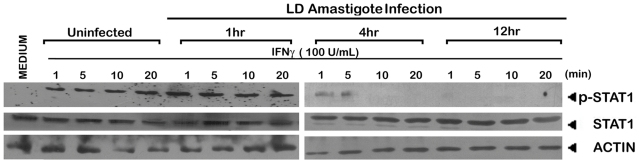
Suppression of IFNγ signaling initiation in LD amastigote infected MØs. The MØs were infected with/without LD amastigotes for indicated time periods as described in *Material and Methods* followed by treatment with 100 U/ml of rIFNγ for varying time durations. Expressions of phosphorylated STAT1 and whole STAT1 were measured in whole cell lysates via western blot using the same membrane. Equal loading was verified by immunoblotting of actin.

As the infection in vivo propagates through amastigotes which lack LPG [53], we asked the question if their role in IFNγ signaling inhibition was different from that of promastigotes. The result depicted in Figure 9 demonstrates that for all the tested time points of amastigote infection, deactivation of the IFNγ signaling takes place as early as 5 min post-IFNγ stimulation. The result was consistent with previous report [55] of immediate deactivation of early IFNγ signaling at the level of JAK2 phosphorylation in LD amastigote infected primary human MØ, post 10 min of rIFNγ stimulation.

Collectively the above results strongly imply that membrane cholesterol is critically involved in the initiation of IFNγ signaling, probably by direct modulation of IFNγ receptor functionality.

### IFNγR1 peptide containing CRAC motif exhibits preferential association with cholesterol

**Figure 10:**
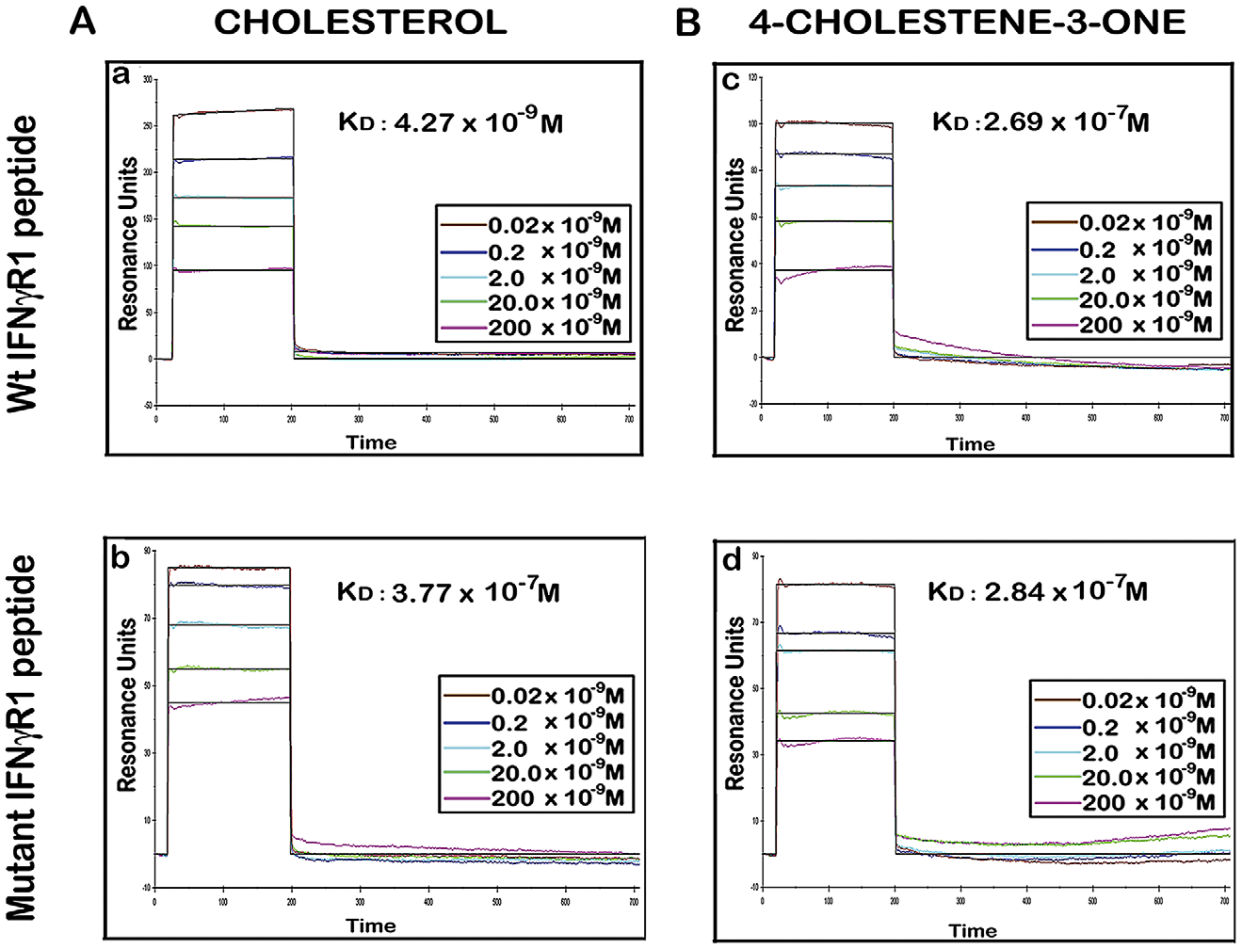
Binding analyses of specific IFNγR1 derived peptides to liposomal cholesterol and 4-cholestene-3-one by SPR. **A**, Sensorgrams of peptide binding with liposomal formulation of cholesterol (a) wild type IFNγR1 peptide and (b) mutant peptide. **B**, Sensorgrams of peptide binding with liposomal formulation of cholesterol analogue 4-cholestene-3-one (c) wild type IFNγR1 peptide and (d) mutant peptide. Respective K_D_ values obtained from global fit of all the graphs are denoted inside. Insets showing the concentrations of peptide used. The black curves in all sensorgrams are the fitting curves using models from BIAevaluation 4.1. Data represents one of two repeat experiments.

There are number of reports on the existence of a common cholesterol recognition/interaction amino acid consensus (CRAC) motif in various proteins [56] including several membrane proteins that directly interact with cholesterol [57]. Very well characterized CRAC motif conforms to the pattern (L/V)-X_1-5_-Y-X_1-5_-(R/K), in which X_1-5_ represents between one to five residues of any amino acid [56], [57]. As an initial effort to explore the possibility that the functionality of constitutively raft associated IFNγR1 protein is directly modulated by the interaction of cholesterol, we tried to detect the cholesterol recognition motif in it. Motif analysis of IFNγR1 indeed identified two CRAC sites within the IFNγR1 subunit protein (Figure S4). Multiple sequence alignment showed it to be highly conserved because the domain is present in many of the homologous proteins in different species (Table S1) nullifying its chance occurrence in murine IFNγR1. As denoted by Jamin et al. the CRAC motif is typically located adjacent to a transmembrane α-helix that positions this sequence within the nonpolar core of the bilayer, where it can form contacts along the length of the cholesterol molecule [58]. Sequence analysis of IFNγR1 predicted the localization of the CRAC motif *(−269 to −280*) within a transmembrane α-helical portion (Figure S5). Accordingly, we designed a peptide reproducing the CRAC motif of IFNγR1 protein (*−269 to −280*) to analyze the binding of the motif with cholesterol liposome. It should be noted that the tyrosine residue in this motif has been shown by mutational analysis to be critical for cholesterol binding in other proteins [58]. Therefore, another peptide was designed to introduce substitution at positions *275* and *277* to replace tyrosine, intended to disrupt/attenuate the binding of the peptide with cholesterol . We used surface plasmon resonance (SPR) to examine the binding of IFNγR1 peptides to liposomal formulation of cholesterol or its oxidized analogue 4-cholestene-3-one. Binding of cholesterol to the Wt IFNγR1 peptide is indicated by the high association rate (k_a_ = 2.69×10^3^ M^−1^ s^−1^) and affinity (K_D_ = 4.27×10^−9^ M) compared to those of the mutant (k_a_ = 27×10^0^ M^−1^ s^−1^, K_D_ = 3.77×10^−7^ M). The specificity of the cholesterol-IFNγR1 peptide interaction was also evident from the 100 fold lower affinity of the Wt peptide for the cholesterol analogue (Table 1 and Figure 10).

**Table 1:** Summary of Binding parameters of IFNγR1 peptide-sterol interaction.

Sterol	Peptide	k_a_(1/M sec)	k_d_ (1/sec)	K_A_ (1/M)	K_D_ (M)
CHOLESTEROL	Wt IFNγR1	2.69×10^3^	1.15×10^−5^	2.34×10^8^	4.27×10^−9^
	Mutant IFNγR1	27.0×10^0^	1.02×10^−5^	2.65×10^6^	3.77×10^−7^
4-CHOLESTENE-3-ONE	Wt IFNγR1	37.8×10^0^	1.02×10^−5^	3.71×10^6^	2.69×10^−7^
	Mutant IFNγR1	35.7×10^0^	1.01×10^0^	3.53×10^6^	2.84×10^−7^

Kinetic and affinity data from SPR analysis of the interaction between different IFNγR1 peptides and sterols. The binding parameters were obtained from global fit of the curves for each experiment.

### Enhancement of IFNγ induced intracellular parasite killing upon liposomal delivery of cholesterol in parasitized MØ

**Figure 11:**
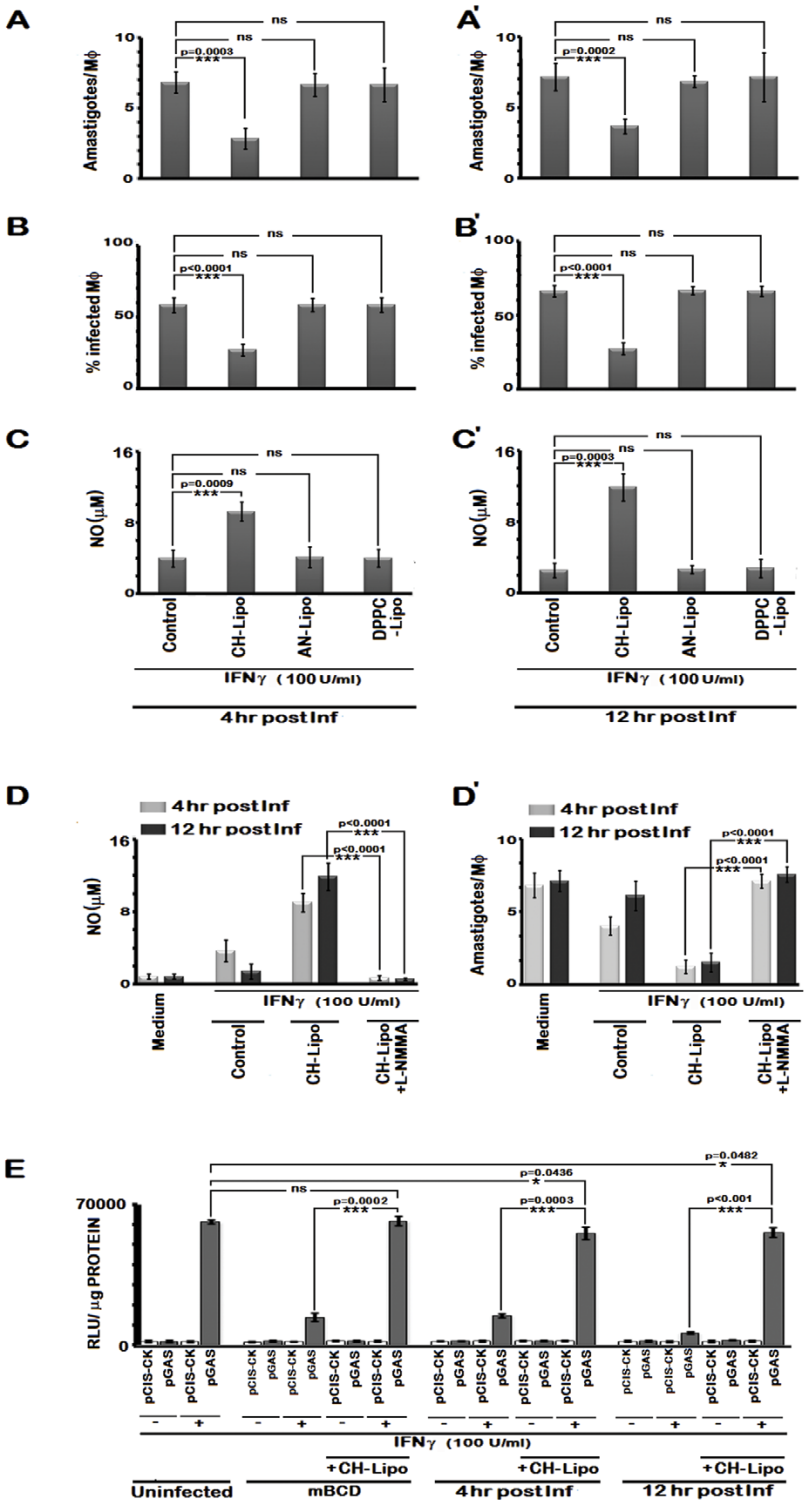
Cholesterol treatment restores IFNγ induced leishmanicidal effects in LD-infected MØs despite prolonged infection. Infected RAW 264.7 cells were left untreated or treated with CH-Lipo or AN-Lipo or DPPC-Lipo followed by stimulation with 100 U/ml of rIFNγ for 24 hours at 4^th^ hour post infection (**A, B, C**) or 12^th^ hour post infection (**A', B', C'**) and the number of intracellular amastigotes per MØ, % infected MØs and nitrite concentration (µM) were determined. Data are representative of three independent experiments. **D**, Pretreatment with inducible nitric oxide synthase inhibitor blocks IFNγ induced leishmanicidal effect in CH-Lipo treated infected MØs. RAW 264.7 cells were infected with LD at m.o.i of 10, for 6 hours. MØs were then washed and either treated with CH-Lipo or left untreated as infected control. Subsequently, at indicated timepoints postinfection, MØs were stimulated with or without 100 U/ml of rIFNγ for 24 hours in presence or absence of 5 µM L-NMMA. Cell free supernatant was harvested for NO measurement. Data of Nitrite concentration (µM) is representative of three independent experiments. The amastigote numbers of the parallel sets treated similarly was demonstrated in **D'**. **E**, Enhanced IFNγ-bioactivity in CH-Lipo replenished MØs as determined by reporter assay. RAW 264.7 cells were transfected with pCIS-CK negative control vector or IFNγ driven pGAS reporter plasmid. 12 hrs after transfection, cells were either left untreated or treated with mBCD (5 mM, 30 min, 37°C) or infected with LD. Treated cells were then replenished or not with CH-Lipo. Subsequently, mBCD treated cells with or without CH-Lipo reloading were stimulated with or without 100U/ml of rIFNγ eight hours before final harvesting for luciferase activity determination in cell lysates. While the infected cells with or without CH-Lipo treatment were stimulated or not with 100 U/ml of rIFNγ at indicated timepoints post infection. Eight hours later, luciferase activities in cell lysates were measured as described in *Materials and Methods*. The data represents the relative luciferase activity normalized to per microgram of protein content. Data is representative of three independent experiments.

We assessed whether cholesterol repletion could restore IFNγ induced leishmanicidal effects of the infected MØs. The results depicted in Figure 11 reveal that IFNγ stimulation of the cholesterol repleted MØs mediated effective restoration of the parasiticidal effects independently of the status of infection progression. The data (Figure 11 A–C) showed 2.41 fold (p = 0.0003 vs infected control) and 2.16 fold (p<0.0001 vs infected control) reduction respectively in the intracellular amastigote number and percent of infected cells, when stimulated by IFNγ at 4 hr post infection, along with 2.51 fold (p = 0.0009) enhanced generation of NO. The observations of parasiticidal effects and NO production were remarkably similar in MØs stimulated with IFNγ at later time points of infection (Figure 11 A', B', C'), suggesting an effective reversal of the IFNγ unresponsiveness of the infected MØs. As anticipated, AN-Liposome or DPPC-Liposome treatment of the LD infected MØs failed to reinstate the MØ responsiveness to IFNγ stimulation.

To ascertain the direct correlation of cholesterol mediated IFNγ signaling with NO production and consequent leishmanicidal effect without involvement of any secondary mediator, L-NMMA was added to cholesterol treated cells. This indeed completely abrogated the enhanced NO production and amastigote killing potential of cholesterol replenished MØs in response to IFNγ (Figure 11 D, D').

We finally assessed the generalized biological response of cholesterol promoted IFNγ signal enhancement in MØs. Analysis of the IFNγ induced transcriptional activity by GAS driven luciferase reporter activity in cholesterol-supplemented systems of CH-Liposome treated LD infected cells and mBCD treated cells demonstrated enhanced bioactivity of IFNγ, as manifested by higher GAS-Luc expression. This suggests that efficient MØ activation could be achieved by cholesterol treatment of deactivated MØ systems.

## Discussion

Our study reinforces the previous findings that LD-MØs are refractory to IFNγ mediated signaling, as evident from the inability to clear intracellular parasites (Figure S1). This was due to defective IFNγ signaling as apparent from a number of functional assays such as defective downstream signaling such as JAK2 phosphorylation (Figure S3A) and IFNγ driven GAS-Luc activity (Figure S3B) under parasitized condition. Interestingly enough, the defect is not due to non-availability of IFNγR on the cell surface (Figure S2A) or inability of the ligand to bind to the cognate receptor as there was a comparable binding affinity (K_D_) in terms of binding of ^125^I-IFNγ between normal and infected conditions (Figure S2B). It was also evident that LD-MØ did not show any generalized defect as IL-10 mediated STAT3 phosphorylation (Figure S3C) and internalization of ^125^I-IFNγ remained unaffected (Figure S2C).

The ligand receptor interaction of IFNγ-IFNγR1 is a sequential multistep process which involves the binding of bivalent IFNγ to the extracellular domain of the cognate receptor subunit of IFNγR1 in a ratio of 1∶2 [59], [60]. This provides the binding scaffold for IFNγR2, the second subunit of the receptor complex [61], [62]. The receptor subunit assembly is unlikely to occur randomly at the plasma membrane of the stimulated cells but is confined to specific cholesterol rich membrane microdomains termed rafts. This can be inferred from indirect evidences derived by the use of cholesterol quenching drugs, which show perturbation of IFNγ signaling by depletion of membrane cholesterol [14]-[16].

The defective IFNγ signaling in LD-MØs was not due to LPG because treatment of normal MØs with exogenous LPG showed neither decrease in fluorescence anisotropy (FA) nor altered IFNγ mediated promoter activity (Figure 8). The defective signaling induced by promastigote mediated infection could also be reproduced with LPG deficient amastigotes (Figure 9); implying that the effect is independent of LPG despite the intercalation of LPG into membrane raft (Figure 8B and ref [54]) and increased the total membrane rigidity (Figure 8A and ref [54]).

From the fractionation study (Figure 2) and confocal microscopy (Figure 3), it was evident that IFNγR1 is raft associated whereas IFNγR2 is largely non-raft associated. Upon ligand driven stimulation, IFNγR2 moves to the raft fractions in normal condition (Figure 2). During infection, restraint in IFNγR2 recruitment in raft fraction even in the presence of IFNγ, suggests that the association of IFNγ-R1 and -R2 in LD-MØ is defective. This observation was further corroborated from co-IP studies (Figure 1B) and confirmed by FRET analysis (Figure 1A). An elegant study showed that the diffusion coefficient of both GPI linked and native I-E^k^ are dependent on cholesterol concentration [63]. Our study also showed that membrane cholesterol decreases in LD-MØ as a function of time (Figure 5A). Our results thus support the notion that the decreasing membrane cholesterol due to the presence of intracellular parasites affects the stimulation dependent mobility of IFNγR2 into rafts as shown in the case of T cell receptors at the plasma membrane [64].

It was also observed that stimulation of normal MØs with IFNγ causes clustering of the entire IFNγ signaling complex in the membrane raft (Figure 4) which was absent under parasitized condition. The effect caused by the intracellular parasites was mimicked by quenching of cholesterol from the membrane by mBCD and restored by liposomal delivery of cholesterol, as evident from FRET analysis (Figure 7B) and fractionation studies (Figure 7A). This observation indicates that intracellular parasites may cause the above defects by quenching cholesterol from the membrane. The specificity of cholesterol in inducing effective IFNγ inducible receptor interaction was assessed by supplementation of the LD-MØs with the oxidized cholesterol analogue 4-cholestene-3-one (Figure 5B, 6A, 6B), which is known not to induce lipid ordered domain or raft formation due to its head group oxidation and differential partitioning behavior compared to cholesterol [65], [66], [23]. The results imply that the oxidized cholesterol analogue could not induce any productive interaction between the receptor subunits of IFNγR1 and IFNγR2, substantiating an obligatory role of cholesterol in maintaining the functional integrity of IFNγR. The membrane packing of cholesterol is supported by a hydrogen bond between the cholesterol OH group and the amide bond of sphingolipids [49]; this interaction is presumably absent in the case of the oxidised analogue because of the lack of OH group.

To assess whether increasing the membrane rigidity could restore the propensity of IFNγR1 and IFNγR2 subunit interaction, we supplemented the cell with DPPC-liposomes (Figure 5B, 6A, 6B) to deliver saturated phospholipids, which increased the membrane rigidity very efficiently. Intriguingly and paradoxically, the increased membrane viscosity resulting from the delivery of DPPC (Figure 5, 6) or LPG (Figure 8), as evident from fluorescence anisotropy values, was insufficient to potentiate IFNγ induced receptor subunit interaction. Collectively these results suggest a direct role of cholesterol in stabilizing the IFNγR interactions, rather than an indirect effect of bulk membrane rigidity to increase the chance interaction of the receptor subunits by random proximity.

Membrane proteins such as G protein coupled receptors, transporters, or ion channels have been shown to be localized or enriched in lipid rafts. The role of cholesterol in receptor conformational stabilization has been reported by several groups, where stability of the active conformation of the receptors depends on the presence of cholesterol with specific molecular interaction between protein and adjacent cholesterol, such as cholecystokinin (type B), oxytocin and nACh receptors [18]. nAChr is reportedly shown to be stabilized in its activated form by a direct action of cholesterol [19]. There are spectroscopic analyses showing that variation in cholesterol concentration can perturb protein secondary structure; this has been shown in photosystem II [67].

Because of nonavailability of pure IFNγR1 receptors (available in the form of chimera with Fc receptor, which itself contains cholesterol binding motif) we could not study the conformation and determine kinetic values of IFNγR1 in association with cholesterol-liposome/analogue-liposome.

As there are reports of cholesterol binding CRAC motifs [(L/V)-X_1-5_-Y-X_1-5_-(R/K)] [57] in a number of membrane proteins, the presence of such a motif in IFNγR was searched for. Sequence analysis of IFNγR1 showed their presence in the transmembrane domain. Kinetic studies revealed conspicuous interaction of such motifs with cholesterol liposomes, but not with analogue liposomes, which differed by two orders of magnitude (K_D_: 4.27×10^−9^ M versus 2.69×10^−7^ M; Figure 10). Consistent with previous reports, the interaction is tyrosine dependent as the tyrosine substituted peptide failed to interact with cholesterol since the association rate was very slow and/or the affinity for cholesterol was 100 fold reduced. These observations, thus clearly denote that *Leishmania* parasites during their intracellular life cycle by modulating membrane cholesterol, exert significant effect on the IFNγR function with subsequent modification of IFNγ mediated intracellular parasite killing.

These results thus represent the first evidence of interaction of cholesterol and cholesterol binding motif in IFNγR1. It corroborates all the previous reports [14]–[16] of cholesterol dependent IFNγR1 functional regulation. One would expect cholesterol-liposome to show leishmanicidal effect in association with IFNγ. Indeed this study clearly showed such effect coupled with NO generation in LD-MØs upon treatment with the above combination. The effect was cholesterol specific because the leishmanicidal effect was not observed with analogue liposome or with DPCC-liposome. This observation lends credence to our previous reports showing strong therapeutic role of liposomal-cholesterol in infected hamster model [23].

Survival strategy of the parasite LD might as well include specific degradation and/or negative regulation of expression of protein molecules of the infected cells such as STAT1 [68] and IFNγR1 [69] - two important components of the IFNγ signaling pathway. The lack of reproducibility of these results in our system is perhaps attributable to the difference in cell system or parasite strains used for infection.

The mechanism by which LD modulates membrane cholesterol is yet unknown. Given the fact that dynamic equilibrium between the pools of free membrane cholesterol and cytoplasmic droplets of cholesterol esters is very tightly controlled [70], diminished membrane cholesterol in LD-MØs, may result from overall decrease in total cellular cholesterol. It may be recalled that in VL patients a progressive decline in serum cholesterol as a function of splenic parasite load [26] is indicative of reduced cellular cholesterol since serum cholesterol and total cellular cholesterol are in dynamic equilibrium [71]. Interestingly, in *L. major* infected macrophages decrease in cellular cholesterol is causally related to reduced mRNA level of HMG-CoA reductase, the rate limiting enzyme of cholesterol biosynthetic pathway [72]. Alternatively, different reports [73], [74] also suggest that LD may exploit host cholesterol for its entry and survival, interfere with cholesterol trafficking or may exchange host cholesterol for ergosterol, with biophysical characteristics grossly different than cholesterol. Thus, these contentions warrant further studies and are currently under our investigation.

We would like to emphasize here that we have specifically dealt with the earliest and critical most step of IFNγ signaling occurring within seconds (180 secs) of receptor ligation and demonstrate that LD infection can efficiently impose a block at this step by specific disruption of the receptor oligomerization by lowering the macrophage membrane cholesterol level. It should be mentioned here that IFNγ signaling amplitude depends on the sustained durability of the activation phenomenon and is very tightly regulated by multiple negative regulatory mechanisms which include dephosphorylation of the activated signaling intermediates by inducible activity of phosphoprotein phosphatases [75], several minutes after ligand–receptor interaction. Seminal study by Blanchette et al. show that this basic physiological phenomenon of signal duration restrain is efficiently utilized by the opportunistic parasite *Leishmania* spp., to attenuate the IFNγ signaling amplitude [76] which in turn affect the overall IFNγ bioactivity in LD infected macrophages which likely contribute in part to the inhibition of complete restoration of the IFNγ signaling pattern in cholesterol treated LD infected macrophages (Figure 11A, A', B, B', C, C', E).

Of note, constitutive SHP-1 preassociation with JAK2 in the steady-state of normal cells as reported by various groups [77], [78] denotes an active repression of JAK2 autophosphorylation. Experimental verification (Figure S6) denotes that in cholesterol depleted systems with segregated IFNγ receptor subunits as in established LD infected macrophages, the SHP-1 species remained associated with the JAK2 proteins, preventing its autophosphorylation upon ligand stimulation, which was in sharp contrast to uninfected macrophages where the SHP-1 is released from JAK2 to induce its autophosphorylation within minutes (3 minutes) after ligand stimulation. Interestingly, cholesterol supplementation in LD infected cells reinstated the JAK2 activation profile and release of SHP-1 from JAK2 immediately upon IFNγ stimulation (our unpublished observation, data not shown).

There is a report that *Leishmania* parasites deliver effector proteins into host cells by exosome-based secretion and can modulate host-cell function [79]. Thus it is not unlikely that uninfected MØs in the infected splenic environment may trap parasite derived antigen (s) and display similar defects in IFNγ signaling like infected MØs. It is worthy of mention that the late established phase of VL is marked with increased production of IL-10 [80]. IL-10, which has a prominent IFNγ desensitizing role acting directly at the level of STAT1 deactivation [81], might account for the chronic infection in the presence of high circulating IFNγ in VL, providing an additional negative regulatory step on IFNγ signaling.

The central point that evolved from this study is that intracellular LD quench membrane cholesterol; this in turn affects the lateral mobility of IFNγR2 into the raft, IFNγR1-R2 multimerization and subsequent downstream signaling in response to IFNγ. This defect could be corrected by exogenous delivery of liposomal cholesterol leading to intracellular parasite clearance. Based on this study, it can be reasonably speculated that cholesterol may also influence the conformation of IFNγR1. Thus in VL, cholesterol-liposomal formulation of IFNγ treatment may emerge as a promising therapeutic strategy both in drug-sensitive and -resistant cases as it is directed specifically towards host than the parasites.

## Materials and Methods

### Ethics statement

Use of mice was approved by the Institutional Animal Ethics Committee of Indian Institute of Chemical Biology, India. All animal experimentations were performed according to the National Regulatory Guidelines issued by CPSEA (Committee for the Purpose of Supervision of Experiments on Animals), Ministry of Environment and Forest, Govt. of India.

### Antibodies and other reagents

Anti-mouse IFNγR1 (MAB1026, Clone- 2E2.4) was obtained from RnD systems, anti-mouse IFNγR2 (#559917, Clone- MOB-47), biotinylated anti-rat IgG, biotinylated anti-hamster IgG, Flottilin1, transferrin receptor (biotinylated CD71), antiSTAT1α, Streptavidin-PE and recombinant mouse IFNγ were purchased from BD Pharmingen, San Jose, California. Anti-LPG antibody (CLP003A, Clone- CA7AE) was from Cedarlane, Ontario, Canada. FBS, RPMI 1640 medium, M199 medium, Penicillin-streptomycin and sodium bicarbonate were obtained from Invitrogen Life Technologies. Cholesterol, HEPES, 2-β ME (beta mercaptoethanol), paraformaldehyde, ponceau S, FITC conjugated cholera toxin B subunit (CTxB-FITC), L-NMMA, 1, 6-diphenyl-1, 3, 5-hexatriene (DPH) and Western Blocker Solution for HRP detection systems were purchased from Sigma-Aldrich (St. Louis, MO). Prestained protein molecular weight marker, Bradford protein assay reagent and Laemmli buffer were from Bio-Rad, Hercules, CA. Hybond ECL nitrocellulose membrane and Hypercassette were from Amersham Biosciences. pJAK1, pJAK2, p-Tyr were purchased from Santa Cruz Biotechnology. JAK1, JAK2 (Santa Cruz Biotechnology) were kind gift from Dr. Santu Bandyopadhyay, IICB (Kolkata, India). Streptavidin-HRP was purchased from Zymed Laboratories. Phosphatidylcholine (PC) (egg lecithin) and 1, 2-dipalmitoyl-sn-glycero-3-phosphocholine (DPPC) was from Avanti Polar Lipids, Inc., Alabaster, Alabama and 4-cholestene-3-one was purchased from ICN (Irvine, CA). All the amino acids were purchased from Novabiochem, Merck. TFA, EDT, thioanisole, TIS were purchased from Merck, Germany.

### Parasite maintenance

LD strain AG83 (MHOM/IN/1983/AG83), originally obtained from an Indian kala-azar patient [82], was maintained in Golden hamsters as described previously [83]. Promastigotes obtained after transforming amastigotes from the spleen of infected animals were maintained in culture in Medium 199 supplemented with 10% FCS at 22°C. The culture was replenished with fresh medium every 72 h.

### Cell lines and preparation of peritoneal exudates cells

Murine MØ-like tumor cell, RAW 264.7 was obtained from American Type Culture Collection. Cells were maintained in complete medium at 37°C with 5% CO_2_ in a humidified atmosphere.

BALB/c mice were obtained from the animal facility of the institute and were housed under conventional conditions, with food and water ad libitum. BALB/c mice were injected with 2 ml of 4% starch i.p. The peritoneal cells (PEC) were harvested 2 days after the starch injection and were rested for 24 h before any treatments on these cells. In the meanwhile, the nonadherent cells were washed out to generate pure adherent populations.

### Infection of PECs and RAW 264.7

PEC and RAW 264.7 cells were allowed to adhere for 4 hr at 37°C in presence of a 5% CO_2_ atmosphere, after which the nonadherent cells were removed by gentle washing with serum-free medium. The adherent MØs, after overnight incubation in complete medium, were challenged with LD promastigotes at a MØ to parasite ratio of 1∶10 and incubated further for 6 hr at 37°C. After initial attachment of 6 hr, excess parasites were washed with serum-free medium, and this was considered as the initial most (0 hr) time point of infection and the infection was allowed to progress for the indicated time periods thereafter, followed by rIFNγ treatment with the suboptimal 10 U/ml, optimal 100 U/ml and supraoptimal dose of 1000 U/ml. The cultures were terminated after indicated timepoints and treatments, by washing and immediate fixing with methanol, followed by staining with Giemsa stain and counted under a light microscope (Leica). The parasite load was expressed as the number of amastigotes per MØ and % infected MØs as described earlier [22]. In some experiments freshly prepared lesion derived LD amastigotes were used for infection at a multiplicity of infection (m.o.i) of 10. The dose of 100 U/ml of rIFNγ was chosen for optimum cell-activation as no additional benefit was obtained for the higher dose tested.

### Measurement of NO

Nitric oxide (NO) generation was monitored by using the Griess reaction as described previously [84], and the results were expressed in µM of nitrite accumulation. In some experiments, MØs were cultured in the presence of N^G^-monomethyl-L-arginine (L-NMMA) a competitive inhibitor of inducible NO synthase, added to a final concentration of 5 µM [85].

### Flow cytometry

Membrane expression of IFNγR1 and IFNγR2 were investigated on resting RAW 264.7 cells with incubation of 1×10^6^ cells with anti-mouse IFNγR1 mAb or anti-mouse IFNγR2 mAb for 30 min at 4°C followed by two washes with cold PBS supplemented with 1% FCS and 0.05% sodium azide (PBS-azide). For secondary labeling, cells were stained with biotin-conjugated anti-hamster antibody for 30 min at 4°C and were washed twice with cold PBS-azide and were then labeled with PE-conjugated Streptavidin. After the final wash with cold PBS-azide, cells were fixed with cold 2% paraformaldehyde in PBS. Membrane protein expression was analyzed with a FACSAria II flow cytometer (Becton Dickinson, Mountain View, CA).

### Radioiodinations and binding assays

Radioiodination of rIFNγ was performed as previously described [86]. rIFNγ (5 µg at 0.5 µg/µl) was radioiodinated with 5 µl (500 µCi) of Na^125^I (17 mCi/µg, BRIT, India) in the presence of 25 µl of 0.15 M potassium buffer, pH 7.4, and 10 µl chloramine-T (5 mg/ml) for 2 minutes. After neutralization of the reaction with 10 µl each of sodium metabisulfite (10 mg/ml), potassium iodide (70 mg/ml), and BSA (20 mg/ml), the reaction mixture was chromatographed over a 10-ml Sephadex G-10 column equilibrated with a Tris/NaCl/BSA buffer (10 mM Tris-HCl, pH 7.4, 0.15 M NaCl, and 0.33 mg/ml BSA). Fractions of 500 to 600 µl were collected, and the fraction containing the greatest activity was used for receptor binding studies. The specific activity of ^125^I-IFNγ was 110 µCi/µg of protein. Anti-mouse IFNγR1 (10 µg) was radioiodinated by following the method essentially described in [87], with minor modifications. Briefly, 50 µl anti-mouse IFNγR1 was incubated with 25 µl (500 µCi) Na^125^I (17 mCi/µg, BRIT, India) in the presence of 125 µl of 0.15 M potassium buffer, pH 7.4, and 50 µl chloramine-T (5 mg/ml) for 4 min. The neutralization of the reaction was done as above and readily sieved on a 10-ml Sephadex G-10 column. Fractions of 400 µl were collected, and the fraction containing the greatest activity was used for receptor binding studies. The specific activity of ^125^I -labeled anti-IFNγR1 was in the range of 100–105 µCi/µg of protein.

Saturation binding assays were performed as previously described with brief modifications [10]. To enumerate receptor numbers, ^125^I-IFNγ was added to final concentrations of 5–800 pM to 2×10^7^ cells per ml of RPMI medium containing 2% fetal bovine serum in duplicates. After 180 min of incubation at 4°C, cells were washed thoroughly with ice cold PBS. The radioactivities associated with separated cell pellet and the supernatant were quantified in a scintillation counter (Packard) to determine free and bound ^125^I-IFNγ respectively. To determine nonspecific binding, the amount of ^125^I-IFNγ bound to the cells in the presence of a 200-fold excess of unlabeled ligand was determined. Specific binding was calculated by subtracting nonspecific binding from the total radioactivity bound. After correcting for nonspecific binding, data of ^125^I-IFNγ saturation binding experiments were fitted to a one-site binding hyperbola and analyzed with non-linear regression analysis to determine K_D_ and Bmax values using GraphPad Prism 5.0 (GraphPad, San Diego, USA) to obtain parameters of equilibrium binding, where Bmax denotes the maximal density of receptor sites and K_D_ denotes the radioligand equilibrium dissociation constant. In addition, ^125^I-IFNγ saturation binding data were displayed using Scatchard plots where the X-axis is specific binding (Bound) and the Y-axis is specific binding divided by free radioligand concentration (Bound/Free) [88].

### Ligand dependent and independent internalization assay

^125^I-labeled murine IFNγ and ^125^I-labeled anti-mouse IFNγR1 were used respectively to determine the ligand induced and constitutive bulk flow endocytosis mediated IFNγR internalization and were performed as previously described [11], [89] with minor modifications. For internalization studies, 2×10^6^ cells were replenished with ice-cold RPMI containing 2% fetal calf serum. Parallel wells received either ^125^I-labeled IFNγ or ^125^I-labeled anti-mouse IFNγR1 and were incubated for 180 minutes in 4°C, after which unbound radioactivity was removed with brief wash with ice cold PBS and incubations were continued in 37°C medium without ligand to allow internalization to occur. At intervals thereafter, cells were immediately added with ice cold PBS and transferred to ice, to stop internalization. Membrane-associated ligand or antibody was removed from the cells with low pH acid wash buffer (0.2 M acetic acid, 0.5 M NaC1, pH 2.5, [90]). Internalized ligand or antibody was determined by solubilization of the acid-treated cells in 1 M NaOH. Radioactivity was measured with a scintillation counter (Packard). The amount of radioactivity remaining with cells that had been kept at 4°C all along before acid treatment was taken to represent ‘nonspecific’ acid-resistant radioactivity and was subtracted from the values obtained from the 37°C incubations to provide an estimate of specific ligand internalization. The data were fitted to linear regression analysis program in GraphPad Prism 5.0 (GraphPad, San Diego, USA) to obtain the rate of internalization by following the methods of Liu et al. [91].

### Cell lysis and western blotting from whole cell lysates

Cell lysis and western blotting were carried as described previously [92] with fewer modifications. In general, uninfected or LD infected RAW 264.7 cells were treated with or without 100 U/ml of rIFNγ for 3 min, 37°C. After washing twice with ice-cold PBS, cells were lysed in 1X RIPA Buffer from Cell signaling technology, Danvers, MA (20 mM Tris-HCl pH 7.5, 150 mM NaCl, 1 mM Na_2_EDTA, 1 mM EGTA, 1% NP-40, 1% sodium deoxycholate, 2.5 mM sodium pyrophosphate, 1 mM beta-glycerophosphate, 1 mM Na_3_VO_4_, 1 µg/ml leupeptin, with recommended addition of 1 mM PMSF immediately before use). In some experiments, cells were treated with 10 ng/ml of rIL-10 for 15 min, 37°C [93]. Protein concentration was determined using the Micro BCA protein assay kit (Pierce Chemical Co. Rockland, IL). Proteins (20 µg/lane) were separated by SDS-PAGE on a 10% gel under reducing conditions and electrotransferred to nitrocellulose membranes (HYBOND ECL, Amersham, Piscataway, NJ) in a transfer buffer consisting of 20 mM Tris-HCl, 150 mM glycine, and 20% methanol. Membranes were incubated at 4°C overnight in blocking buffer (Western Blocker Solution, Sigma). Primary and secondary Abs were diluted as recommended by the manufacturer in blocking buffer and incubated with the membranes for 1 hr, at room temperature with five washes in between, with wash buffer (20 mM Tris, 500 mM NaCl, pH 7.4). Detection of HRP-conjugated Abs was performed using SuperSignal West Pico Chemiluminescent Substrate (Pierce, Rockland, IL).

### Transfection and transient expression of luciferase reporter gene constructs

Interferon gamma driven luciferase reporter activity was essentially done as in ref. [94]. RAW 264.7 cells were transfected with the pGAS cis-reporter plasmids or the pCIS-CK negative control plasmid by electroporation [95] by Genepulser X cell (Bio-Rad, Hercules, CA). Cells from three electroporations were pooled to eliminate differences between individual transfections and the mixture was then equally divided among the wells of a 24-well cluster dish (Corning CoStar Corp., Cambridge, MA). They were next allowed to adhere to the substratum for 4 hr before the medium was changed. After 24 hr (which was needed to reduce the background levels of luminescence), the medium was again changed and were either infected with LD or left uninfected. After the initial attachment of the parasites with the cells for 6 hr, the cells were washed and the infection was allowed to progress for the indicated time periods. Thereafter, cultures were incubated for 8 hr with medium alone or medium that contained 100 U/ml IFNγ. The cells were harvested in 300 µl of passive lysis buffer (Promega Biotech, Madison, WI), and 50 µl aliquots of the clarified extracts were used to assay luciferase activity using Luciferase assay kits/reagents from Promega Biotech according to the manufacturer's protocols. Luciferase activity was normalized to the levels of the protein content. The average is reported along with the standard deviation of the mean. The pGAS cis-reporter plasmid and the pCIS-CK negative control plasmid were purchased from Stratagene, La Jolla, CA.

### Liposome preparation

Unilamellar liposomes were prepared from PC with either cholesterol or an analogue of cholesterol (4-cholesten-3-one) at a molar ratio of 1∶1.5 as described in ref. 22, 23. For liposome preparation with DPPC, we followed the methods of Giraud et al. [96]. DPPC (13.8 mg) was dissolved in a chloroform-methanol (9∶1 v/v) mixture. The solvent was removed by evaporation under vacuum, followed by overnight drying of the resulting film under vacuum to remove the residual solvents. The dry film of DPPC was rehydrated in PBS, pH 7.4, then alternately heated in a water bath at 60°C and mixed via a vortex mixer at room temperature for 5 min to allow full hydration of the phospholipid and finally sonicated using an ultrasound 2 mm tip in microprobe sonicator. According to the conventional procedure, repetitive 30-sec cycles of sonication/ice cooling were performed. Samples were briefly centrifuged to remove titanium particles.

### FRET analysis and DyLight labeling of primary antibodies

For fluorescence resonance experiments, anti-mouse IFNγR1 and anti-mouse IFNγR2 were conjugated to DyLight488 or DyLight594 (Pierce; N-hydroxy succinimide (NHS) ester-activated fluorescent dyes) respectively, according to the manufacturer's protocol. Naïve or infected MØs were stimulated with/without IFNγ for 3 min, 37°C to induce FRET signals. The stimulation was terminated by immediate transfer of cells to ice and washing with ice-cold PBS. The cells were then fixed with 2% paraformaldehyde/PBS at 4°C. After washing the paraformaldehyde and quenching the excess formaldehyde for 15 min with 0.1% NaBH_4_ in PBS, staining was followed with excess antibodies to respective IFNγR subunits at 4°C, for 1 hr. Following 3 washes with cold buffer, cells were resuspended in PBS. Spectroscopic analysis of cells in a quartz cuvette was done with to 485 nm excitation and recording the emission spectra from 500 nm to 700 nm with emission and excitation slit of 2.5 nm. FRET efficiency (E) was obtained from the measured fluorescence intensity of the donor (Dylight488- IFNγR1) at its maximal emission wavelength (519 nm) in the presence and absence of the acceptor (Dylight594- IFNγR2) using the equation with E (%)  =  (1 – I_da_/I_d_), where I_da_ and I_d_ are the fluorescence intensities in the presence and absence of acceptor, respectively [97], [98].

### Co-Immunoprecipitation and immunoblotting

For co-immunoprecipitation, experimental cells, treated as indicated in the figure legends, were aspirated off the medium and washed with ice cold PBS, lysed in Cell Lysis Buffer (1X), Cell signaling technology (20 mM Tris pH 7.5, 150 mM NaCl, 1 mM EDTA, 1 mM EGTA, 1% Triton X-100, 2.5 mM sodium pyrophosphate, 1 mM β-glycerophosphate, 1 mM Na_3_VO_4_, 1 µg/ml Leupeptin with 1 mM PMSF added immediately prior to use). After 10 min on ice, lysates were collected by scraping, sonicated for 15 s (0.3 s bursts), centrifuged at 10, 000 r.p.m. for 10 min at 4°C and the supernatant was recovered. Total protein concentrations were determined using the Micro BCA protein assay kit (Pierce Chemical Co.) with BSA as standard. Lysates were pre-cleared using Protein A/G plus Agarose beads (Pierce Chemical Co.). 200 µl of precleared supernatants were incubated overnight at 4°C with specific primary Ab to IFNγR1 or control unrelated whole rabbit IgG, and the immune complexes were collected on 20 µl of 50% bead slurry Protein A/G Plus-Agarose (prewashed and preblocked with 2% BSA for 2 hour, at 4°C in 25 mM Tris, 150 mM NaCl; pH 7.2) for 2 h, at 4°C, with gentle shaking. Precipitates were washed 5 times with 500 µl of 1X Cell Lysis Buffer, kept on ice during washes and microcentrifuged for 60 seconds at 4°C at 2500 x g, each time. The pellet was resuspended with 20 µl of 2X Laemmli sample buffer [(Bio-Rad, Hercules, CA), 62.5 mM Tris-HCl, pH 6.8, 25% glycerol, 2% SDS, 0.01% Bromophenol Blue, 5% β-mercaptoethanol], vortexed for 30 seconds and boiled for 5 min before resolving in SDS-PAGE gels and probed with antibodies as detailed in the figure legends and detected as previously stated.

### Analysis of membrane fractions on floatation gradients

Total crude cell membranes were isolated as described in ref. [99]. Cells were homogenized in 1 ml of buffer [10 mM Tris-HCl (pH 7.4), 1 mM EDTA, 200 mM sucrose] and protease inhibitor mix (Roche Diagnostics, Mannheim, Germany)]. The nuclei and cellular debris were removed by centrifugation at 900 x g for 10 min at 4°C. The resulting supernatant was centrifuged at 110,000 x g for 75 min at 4°C to obtain the crude membrane pellet. Purification method of detergent-soluble and -insoluble fractions was adapted from ref. [100], with very few modifications. The crude membrane pellet was solubilized in 1 ml of Lysis buffer (Caveolae/ Rafts Isolation Kit, Sigma) containing 1% Triton X-100 and was agitated end to end for 30 min at 4°C. The sample was mixed with OptiPrep and to a final OptiPrep concentration of 35%. Then the density gradient was made of 5 layers of OptiPrep with 35, 30, 25, 20, and 0%. The volume of each layer was 2 ml except for 1 ml of 0% OptiPrep. Samples were centrifuged at 200,000 x g for 4 h at 4°C. After centrifugation, nine fractions of 1-ml from the top to bottom of the ultracentrifuge tube were collected. As described in manufacturer's instruction, caveolin-1 positive fractions were found in fractions 2–4 (25–35% OptiPrep) counting from the top (data not shown), which were also enriched in GM1 content. Ganglioside GM1 was visualized with an HRP-conjugated cholera toxin B subunit (1∶2000, Sigma) in dot blots with 2 µl sample from each gradient fraction. In some experiments, fractions 2–4 or fractions 7–9 were combined, and are referred to as Triton-insoluble glycolipid enriched membranes (rafts) or Triton-soluble (non-raft) fractions, respectively. Equal volumes (50 µl) of each fraction were analyzed directly by western blotting.

### Confocal microscopy, image processing and quantitation of signal overlap on fixed cells

Experimental cells were grown on glass-coverslips and treated as indicated in figure legends and after final treatment, washed with cold wash buffer. Cells, preblocked in ice cold blocking buffer (1X PBS with 2 % FBS) for 30 minutes in 4°C were stained with anti-mouse IFNγR1 or anti-mouse IFNγR2 in blocking buffer diluted to a predetermined optimal concentration and incubated at 4°C in the dark. The reaction was stopped by adding ice cold wash buffer and washed three times. For staining with secondary Ab, biotin-conjugated Fc specific anti-IgG was added and stained as described above and followed by streptavidin-PE. The cells were counterstained with FITC-CTxB following the protocol described above. The cells were then fixed with 1% paraformaldehyde, mounted with 90% glycerol on a glass slide, and observed under a laser scanning microscope [Leica (Mannheim, Germany) TCS SP2 AOBS system] by using a 63 oil-immersion objective. Double-stained images were obtained by sequential scanning for each channel to eliminate the “cross-talk” of chromophores and to ensure reliable quantification of colocalization [101]. Images were acquired and processed for colocalization analysis in TIFF format. Colocalization was estimated using Pearson's correlation coefficient [102] analyzed by ImageJ software (rsbweb.nih.gov/ij/).

### Immunoprecipitation and immunoblotting from purified raft and non-raft fractions

Total IFNγR1 or engaged IFNγR1 were immunoprecipitated from pooled ‘rafts’ fraction (2, 3 and 4) and the ‘non-raft’ fraction (7, 8 and 9) as described previously [103], [104] with minor modifications of both. Cells were stimulated for 3 minutes at 37°C with biotinylated rIFN-γ [Recombinant murine IFN-γ was conjugated to EZ linked N-hydroxysulfosuccinimide-biotin (Pierce, Rockford, IL) according to the manufacturer's instruction. Cells were incubated with 0.5 ng/ml biotin-conjugated IFNγ, which is the calculated approximate equivalence to 100 U/ml of bioactive unconjugated IFNγ in our system and gave unaltered stimulation index in terms of STAT1 phosphorylation and pGAS-Luc activity in supportive experiments as with the unconjugated rIFNγ (data not shown)]. Stimulation was terminated by immediate washing with ice cold PBS and harvesting cells in lysis buffers. To immunoprecipitate total amount of IFNγR1 from isolated raft and non-raft fractions, 0.5 ml of the pooled raft and non-raft fractions were added to 0.5 ml of cell lysis buffer, were pre-cleared, and the signaling complex was immunoprecipitated overnight at 4°C with anti-IFNγR1 (10 µg/ml) and the recovery of the pulled immunecomplex was done exactly as described before. Alternatively, engaged IFNγR1 were immunoprecipitated from pooled raft and non-raft fractions with streptavidin-agarose beads. Pooled ‘rafts’ fraction (2, 3 and 4) and the ‘non-raft’ fraction (7, 8 and 9) pre-cleared of non-specific binding proteins by incubation with 50 µl of agarose beads (prewashed and suspended to 50% in 1X cell lysis buffer). Pre-cleared lysates were incubated with streptavidin-agarose beads (50 µl of even suspension of 50% bead slurry ) for 2 hr at 4°C with gentle end over end shaking, washed using our immunoprecipitation protocol and procured from the beads and analyzed as before. Control experiments confirmed that no proteins bound to streptavidin-agarose beads if cells were left untreated with biotinylated IFNγ (data not shown). Where indicated, the IFNγ signaling complex pulled down by immobilized streptavidin-agarose was dissociated in 50 µl of PBS containing 1% SDS by boiling for 10 min and diluted 20-fold with lysis buffer before it was subjected to a second immunoprecipitation using 10 µg/ml of an anti-pTyr Ab. The washing, procurement and immunoblot analysis were done exactly as mentioned before.

### Methyl β-cyclodextrin (mBCD) treatment of cells

For some experiments, cells were treated with mBCD (Sigma-Aldrich, St. Louis, MO) to disrupt lipid rafts. In the initial set of experiments, cells were treated with different concentration of mBCD ranging from 2 to 20 mM in the absence of serum for 30 min at 37°C, after which cell viability was assessed by trypan blue exclusion and the cell membrane cholesterol content and the cell-membrane fluidity were measured (data not shown). Only conditions that allowed for cell viability comparable to mock treatment were used for these studies. It was observed that RAW 264.7 cells maintained the ability to exclude vital dyes such as trypan blue after treatment with 5 mM mBCD though treatment with the same essentially caused an acute depletion of cholesterol content from the cell membrane almost equivalent to the conditions of prevailing in MØ with 12 hr LD infection.

### Measurement of fluorescence anisotropy (FA)

The membrane fluorescence and lipid fluidity of cells were measured following the method described by Shinitzky and Inbar [105]. Briefly, the fluorescent probe DPH was dissolved in tetrahydrofuran at 2 mM concentration. To 10 ml of rapidly stirring PBS (pH 7.2), 2 mM DPH solution was added. For labeling, 10^6^ cells were mixed with an equal volume of DPH in PBS (C*_f_* 1 µM) and incubated for 2 hr at 37°C. Thereafter the cells were washed thrice and resuspended in PBS. The DPH probe bound to the membrane of the cell was excited at 365 nm and the intensity of emission was recorded at 430 nm in a spectrofluorometer. The FA value was calculated using the equation: FA  =  [(I_∥_ -I _⊥_)/ (I_∥_ + 2I _⊥_)], where I_∥_ and I _⊥_ are the fluorescent intensities oriented, respectively, parallel and perpendicular to the direction of polarization of the exciting light [106], [22].

### Measurement of cholesterol content

Sample for cholesterol estimation was essentially prepared following the methods described in ref [107]. The crude plasma membrane fraction was isolated as described above from the experimental cells and suspended in a minimum volume of PBS. An aliquot was used for protein measurement. The rest of the pellet was extracted with 2∶1 methanol/chloroform, followed by 0.5 ml of chloroform and 0.5 ml of water. The methanol/chloroform (lipid phase) layer was dried under vacuum. The dry lipid was suspended in 200 µl of 1X Reaction buffer provided in Amplex Red Cholesterol Assay Kit (Molecular Probes' Invitrogen) and membrane cholesterol quantitation was performed exactly according to manufacturer's instructions.

### Interaction of purified LPG with MØs

A method modified from ref. [108] was employed. Lyophilized lipophosphoglycan (LPG), purified from LD (a kind gift from Prof SJ Turco, University of Kentucky, Lexington, USA), was solubilized in double–distilled water to 1 mM stock and frozen in −20°C as aliquots. Stock solution was diluted in PBS and added to the MØ cultures to a final concentration of 10 µg/ml and was incubated at 37°C in 5% CO_2_ and high humidity for indicated time duration, in each experiment. The cells were washed three times in cell–culture medium with 5% FCS at room temperature to remove unbound LPG followed by washing three times in PBS at 4°C to terminate the experiment.

### Peptide synthesis and purification

Sequence analysis of IFNγR1 protein was done with ScanProsite software (EXPASY TOOLS) to find out consensus motif for cholesterol binding and the peptides were designed accordingly. Peptide designed and synthesized from wild type IFNγR1 protein, reproducing cholesterol binding motif (CRAC motif, residues −*269* to −*280*) was designated as Wt IFNγR1 peptide (269- VILVFAYWYTKK-). Point mutant IFNγR1 peptide (269- VILVFAAWATKK-) reproduces the tyrosine to alanine substituted form of Wt IFNγR1 peptide. The peptides were synthesized on Rink Amide MBHA resin using standard solid phase Fmoc chemistry [109] with a capping step with 5% acetic anhydride and 5% lutidine in DMF after each coupling using PS3 peptide synthesizer (Protein Technologies Inc, USA). Fmoc-amino acids were activated with HBTU in presence of HOBt and DIEA. Peptides were cleaved from the resin and side-chain protecting groups were removed by incubating with 94% TFA, 2.5% EDT, 1.5% thioanisole, 1.5% water, 0.5% TIS for 3 hrs at room temperature after which peptides were precipitated with ice-cold diethyl ether. Peptides were then purified by HPLC (Waters, USA) on a reverse phase µbondapak C-18 column using 0–80% acetonitrile in 0.01% TFA and molecular weight determined by MALDI-TOF/TOF analyzer (Applied Biosystem, USA).

### Binding studies using SPR

Binding experiments were done by surface plasmon resonance (SPR) as described [110], on a BIAcore 3000 system (Biacore AB, Piscataway, NJ). L1 sensor chip (Biacore) with hydrophobically modified dextran layer was used to for SPR experiment to study binding of peptide to immobilized liposomes. Freshly prepared liposome were immobilized on L1 sensor chip up to response unit of 2000–2500 at flow rate of 5 µl/min in PBS (pH = 7.2). To remove any multilamellar structures from the lipid surface, we injected NaOH (50 µl, 10 mM) at a flow rate of 50 µl /min, which resulted in a stable baseline corresponding to the lipid monolayer linked to the chip surface. The negative control BSA was injected (25 µl, 0.1 mg/µl in PBS) to confirm complete coverage of the nonspecific binding sites. After complete association required dissociation time was given to wash loosely bound liposomes and to achieve stable base line. Kinetic experiments were performed with serial dilution of the peptides. Concentration of the peptides used were 0.02 nM, 0.2 nM, 2 nM, 20 nM, 200 nM in PBS (pH = 7.2). Peptides binding measured by observing the change in SPR angle as 90 µl of peptide analyte flowed over the immobilized liposome for 3 min at flow rate 30 µl/min. L1 sensor chip was regenerated using 200 µl of 20 mM CHAPS at flow rate of 50 µl/min. Different dilutions of protein samples in buffer were injected at a flow rate of 30 µl/min. The response was monitored as a function of time (sensorgram) at 25°C. SPR experiments were conducted in duplicate at each concentration to confirm the bindings were repeatable. Multiconcentration data were globally fit, and residuals were calculated and used to assess the goodness of fit with BIAevaluation 4.1 software (Biacore, Piscataway, NJ).

### Statistical variation and presentation

Each experiment was performed thrice, and representative data from one set of these experiments are presented; the interassay variation was within 10%. Two-tailed Student's t test was performed to ascertain the significance of the differences between the means of the control and the experimental groups. We considered values of P<0.05 to be statistically significant. P value <0.001 were considered extremely significant (***), P value ranging between 0.001 to 0.01 were very significant (**), P value 0.01 to 0.05 as significant (*) and P value >0.05 were not significant (ns) (Student's t test). Error bars indicate mean ± SD. Data was analyzed by using Prism 5.0 (GraphPad, San Diego, CA).

## Supporting Information

Figure S1 **Loss of IFNγ response in MØs from very early time point of LD infection.** **A,** Monolayers of freshly plated and adhered mPEC (**A** and **B)** and RAW 264.7 (**D** and **E**) were either left untreated or exposed to promastigotes of *Leishmania donovani* at a multiplicity of infection (m.o.i) 10∶1. After initial attachment of 6 hr, excess parasites were washed and the infection was allowed to progress for the indicated time periods, followed by rIFNγ treatment with suboptimal 10 U/ml, optimal 100 U/ml and supraoptimal 1000 U/ml doses for 24 h. The intracellular parasite number was expressed as amastigotes per MØ and percentage infected cells in the culture. **C** and **F,** Culture supernatants from MØs treated exactly as in **A–E** were harvested and assayed for Nitrite concentration (µM) by Griess reaction as described in *Materials and Methods*. The results shown are from one of three identical experiments which yielded similar results, and represent the mean ± SD of triplicate determinations for each experimental group.
(TIF)

Figure S2 **Unaltered endogenous cell surface IFNγR expression, ligand binding and ligand induced receptor internalization kinetics in LD infected MØs.** **A,** Flow cytometric analysis of surface expression of IFNγR1 and IFNγR2 in RAW 264.7 cell that were left uninfected or infected with LD at a m.o.i of 10 for indicated time periods. Numbers in the plot indicate the mean fluorescence intensity. **B**, Non-linear regression analysis of the binding of IFNγ to the IFNγR protein in normal and LD infected MØs. Uninfected and infected MØs were treated with increasing concentrations of trace labeled 125I- IFNγ for 3 hr at 4°C in the presence or absence of 200 fold excess of cold IFNγ. Scatchard plots (insets) and KD and Bmax values were obtained using the Graph Pad Prism program by analyzing data for specific IFNγ binding. Data represents the mean of duplicate measurements for each concentration of IFNγ. **C**, Rate of ligand induced IFNγR internalization in uninfected and infected RAW 264.7. Cells left uninfected or infected for 4 hr or 12 hr with LD were treated with ^125^I- IFNγ (100 U/ml) for 3 hr at 4°C followed by incubation at 37°C for various time intervals. At the end of incubation, cells were rinsed twice with ice cold PBS and then treated with a low pH buffer (pH 2.5) for 5 minutes at 4°C that removes surface-bound ligand. The acid-inaccessible internalized ligand is presented as a fraction of total cell-associated radioactivity prior to cell transfer to 37°C. For control, we tested the behavior of steady-state internalization of IFNγR by labeling them with ^125^I-IFNγR1 antibody for each experimental set tested. The constitutive and inducible internalization rate was shown in inset and calculated by linear regression analysis in Graph Pad Prism.
(TIF)

Figure S3 **Suppression of IFNγ signaling initiation in LD infected MØs.** **A**, The MØs were infected with/without LD promastigotes for indicated time periods as described earlier, followed by treatment with 100 U/ml of rIFNγ for 3 min. Expressions of phosphorylated JAK2 and whole JAK2 were measured in whole cell lysates via western blot using the same membrane. Densitometric readings represent the ratio of intensity of phosphorylated JAK2 (pJAK2) to JAK2 protein expression per unit area and are represented as arbitrary units (au). Equal loading was verified by immunoblotting of cytoplasmic actin. **B**, RAW 264.7 cells were transfected with either pGAS-Luc or the pCIS-CK negative control plasmid. After 12 hr of transfection, cells were either left uninfected or infected with LD and at indicated timepoints postinfection cells were treated with 100 U/ml rIFNγ for 8 hrs, before final harvestation. Cell lysates were prepared, and luciferase assay was done as described under *Materials and Methods*. Medium control denotes the transfected cells without any subsequent treatments. The values shown in all panels are the average of triplicate measurements in a single experiment and are expressed as relative light units (RLU) normalized to total protein content in each sample. **C**, Assessment of the unrelated JAK-STAT pathway by IL-10 sensitization of LD infected MØs. The MØs were left uninfected or infected with LD promastigotes for indicated time periods as described earlier, followed by treatment with 10 ng/ml of rIL-10 for 15 minutes. Expressions of phosphorylated STAT3 and STAT3 were measured in whole cell lysates via western blot using the same membrane. Equal loading was verified by immunoblotting of actin.
(TIF)

Figure S4 **Scanprosite analysis of mouse IFNγR1 protein sequence (Accession: EDL03452.1 GI: 148671505) denoting the CRAC motifs.** Two CRAC motifs in IFNγR1 protein sequence located within amino acid position −*269* to −*280* and −*393* to −*399* are highlighted in yellow.
(TIF)

Figure S5 **Transmembrane helix prediction result of mouse IFNγR1 protein sequence (Accession: EDL03452.1 GI: 148671505).** The predicted α-helical portion of IFNγR1 protein sequence is denoted as letter ‘H’ in red.
(TIF)

Figure S6 **SHP-1 forms a constitutive complex with JAK2 in macrophages and the release of SHP-1 is IFNγ inducible.** The MØs were infected with/without LD promastigotes for 12 hrs as described earlier, followed by treatment with 100 U/ml of rIFNγ for 3 minutes. Cells were then treated with/without cell permeable crosslinker of DSS, as per manufacture's protocol (Pierce). After cell lysis, protein extracts was immunoprecipitated with anti-JAK2. To verify the identity of JAK2 and SHP-1, immunoprecipitates were separated on polyacrylamide-SDS gel, and immunoblotting was performed with anti-JAK2 antibody or anti-SHP-1 antibody.
(TIF)

Table S1 **Identification of the cholesterol recognition / interacting amino acid consensus pattern.** Alignment of amino acid sequences showing presence of CRAC motif conforming to **-L/V-(X)_1–5_-Y-(X)_1–5_-R/K-** in IFNγR1 proteins in different species.
(DOC)
