# Supplementary figures and images for: Restoration of IFNγR Subunit Assembly, IFNγ Signaling and Parasite Clearance in Leishmania donovani Infected Macrophages: Role of Membrane Cholesterol
Source: PLoS Pathog. 2011 Sep 8;7(9):e1002229. doi: 10.1371/journal.ppat.1002229 (PMC3169561; doi:10.1371/journal.ppat.1002229)

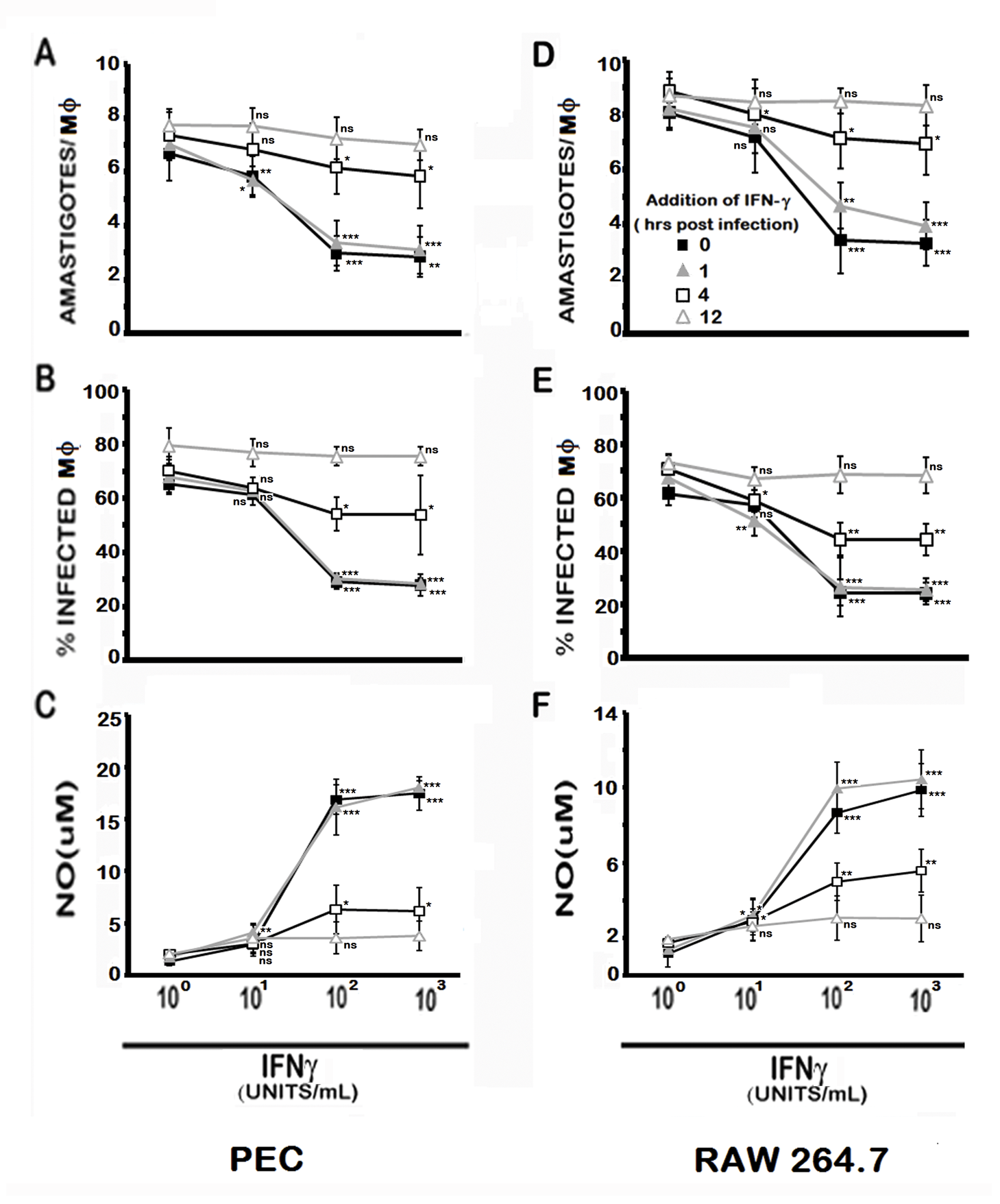

Supplement: Figure S1 — Loss of IFNγ response in MØs from very early time point of LD infection. A, Monolayers of freshly plated and adhered mPEC (A and B) and RAW 264.7 (D and E) were either left untreated or exposed to promastigotes of Leishmania donovani at a multiplicity of infection (m.o.i) 10∶1. After initial attachment of 6 hr, excess parasites were washed and the infection was allowed to progress for the indicated time periods, followed by rIFNγ treatment with suboptimal 10 U/ml, optimal 100 U/ml and supraoptimal 1000 U/ml doses for 24 h. The intracellular parasite number was expressed as amastigotes per MØ and percentage infected cells in the culture. C and F, Culture supernatants from MØs treated exactly as in A–E were harvested and assayed for Nitrite concentration (µM) by Griess reaction as described in Materials and Methods. The results shown are from one of three identical experiments which yielded similar results, and represent the mean ± SD of triplicate determinations for each experimental group. (TIF) [file ppat.1002229.s001.tif]

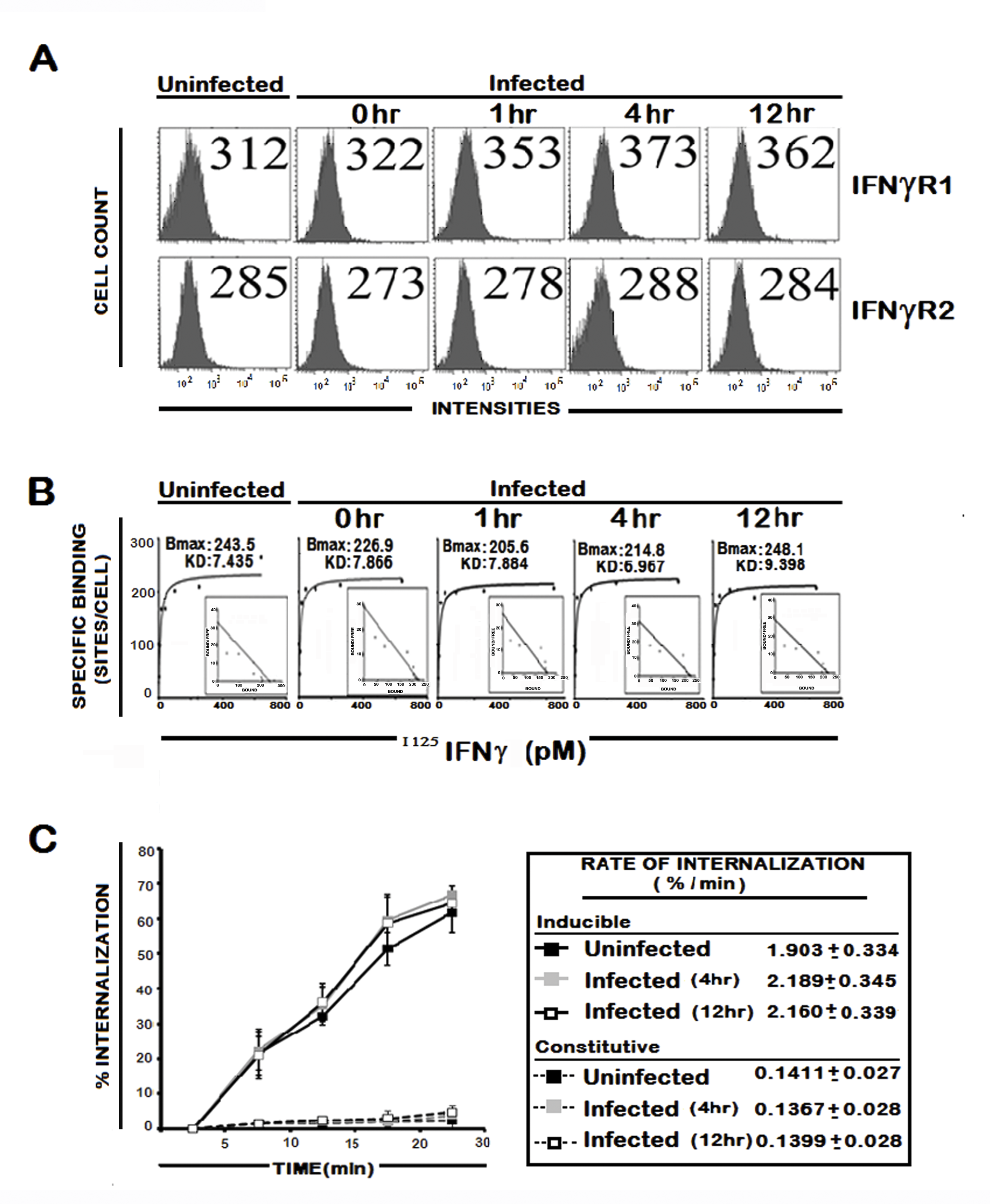

Supplement: Figure S2 — Unaltered endogenous cell surface IFNγR expression, ligand binding and ligand induced receptor internalization kinetics in LD infected MØs. A, Flow cytometric analysis of surface expression of IFNγR1 and IFNγR2 in RAW 264.7 cell that were left uninfected or infected with LD at a m.o.i of 10 for indicated time periods. Numbers in the plot indicate the mean fluorescence intensity. B, Non-linear regression analysis of the binding of IFNγ to the IFNγR protein in normal and LD infected MØs. Uninfected and infected MØs were treated with increasing concentrations of trace labeled 125I- IFNγ for 3 hr at 4°C in the presence or absence of 200 fold excess of cold IFNγ. Scatchard plots (insets) and KD and Bmax values were obtained using the Graph Pad Prism program by analyzing data for specific IFNγ binding. Data represents the mean of duplicate measurements for each concentration of IFNγ. C, Rate of ligand induced IFNγR internalization in uninfected and infected RAW 264.7. Cells left uninfected or infected for 4 hr or 12 hr with LD were treated with 125I- IFNγ (100 U/ml) for 3 hr at 4°C followed by incubation at 37°C for various time intervals. At the end of incubation, cells were rinsed twice with ice cold PBS and then treated with a low pH buffer (pH 2.5) for 5 minutes at 4°C that removes surface-bound ligand. The acid-inaccessible internalized ligand is presented as a fraction of total cell-associated radioactivity prior to cell transfer to 37°C. For control, we tested the behavior of steady-state internalization of IFNγR by labeling them with 125I-IFNγR1 antibody for each experimental set tested. The constitutive and inducible internalization rate was shown in inset and calculated by linear regression analysis in Graph Pad Prism. (TIF) [file ppat.1002229.s002.tif]

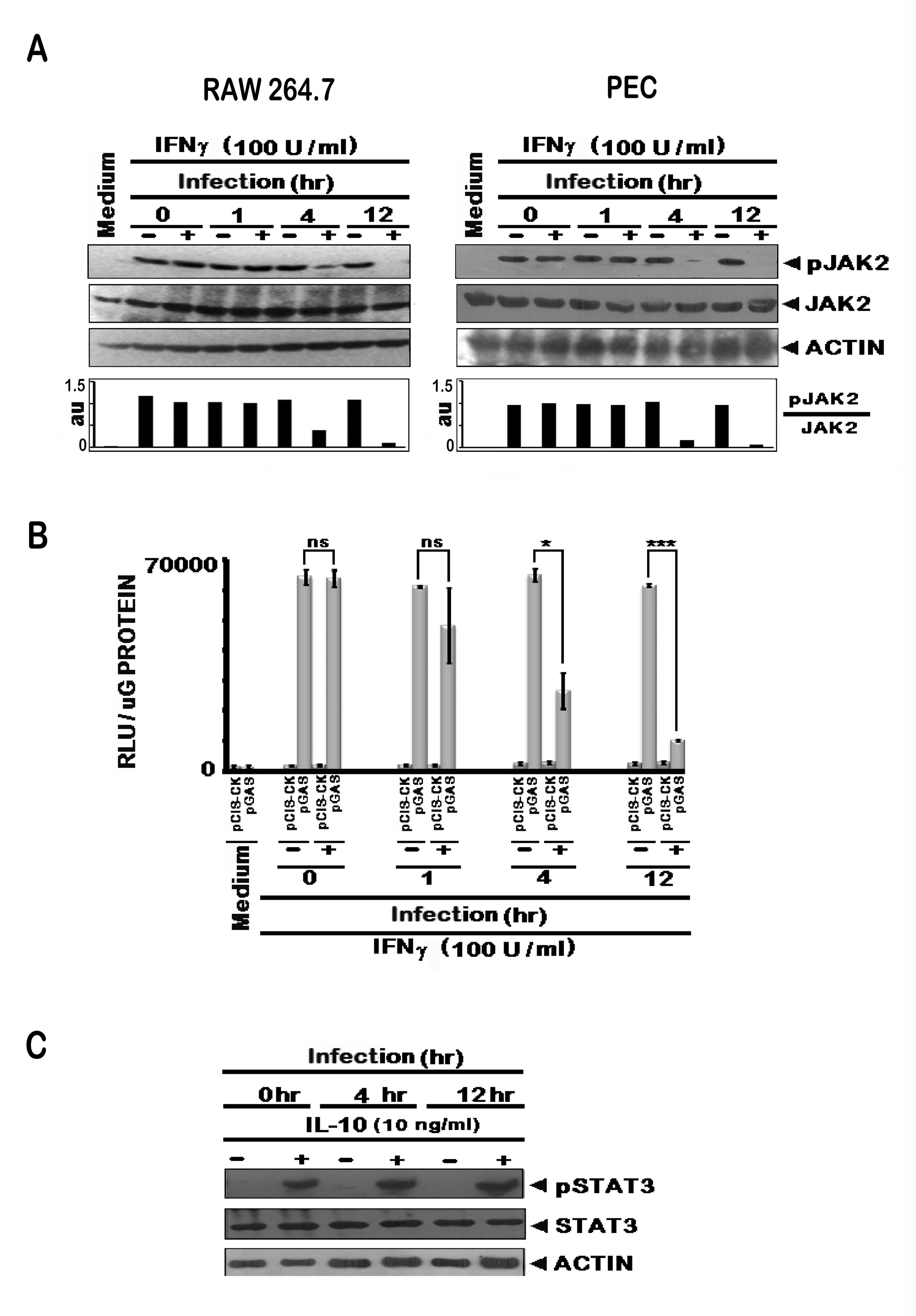

Supplement: Figure S3 — Suppression of IFNγ signaling initiation in LD infected MØs. A, The MØs were infected with/without LD promastigotes for indicated time periods as described earlier, followed by treatment with 100 U/ml of rIFNγ for 3 min. Expressions of phosphorylated JAK2 and whole JAK2 were measured in whole cell lysates via western blot using the same membrane. Densitometric readings represent the ratio of intensity of phosphorylated JAK2 (pJAK2) to JAK2 protein expression per unit area and are represented as arbitrary units (au). Equal loading was verified by immunoblotting of cytoplasmic actin. B, RAW 264.7 cells were transfected with either pGAS-Luc or the pCIS-CK negative control plasmid. After 12 hr of transfection, cells were either left uninfected or infected with LD and at indicated timepoints postinfection cells were treated with 100 U/ml rIFNγ for 8 hrs, before final harvestation. Cell lysates were prepared, and luciferase assay was done as described under Materials and Methods. Medium control denotes the transfected cells without any subsequent treatments. The values shown in all panels are the average of triplicate measurements in a single experiment and are expressed as relative light units (RLU) normalized to total protein content in each sample. C, Assessment of the unrelated JAK-STAT pathway by IL-10 sensitization of LD infected MØs. The MØs were left uninfected or infected with LD promastigotes for indicated time periods as described earlier, followed by treatment with 10 ng/ml of rIL-10 for 15 minutes. Expressions of phosphorylated STAT3 and STAT3 were measured in whole cell lysates via western blot using the same membrane. Equal loading was verified by immunoblotting of actin. (TIF) [file ppat.1002229.s003.tif]

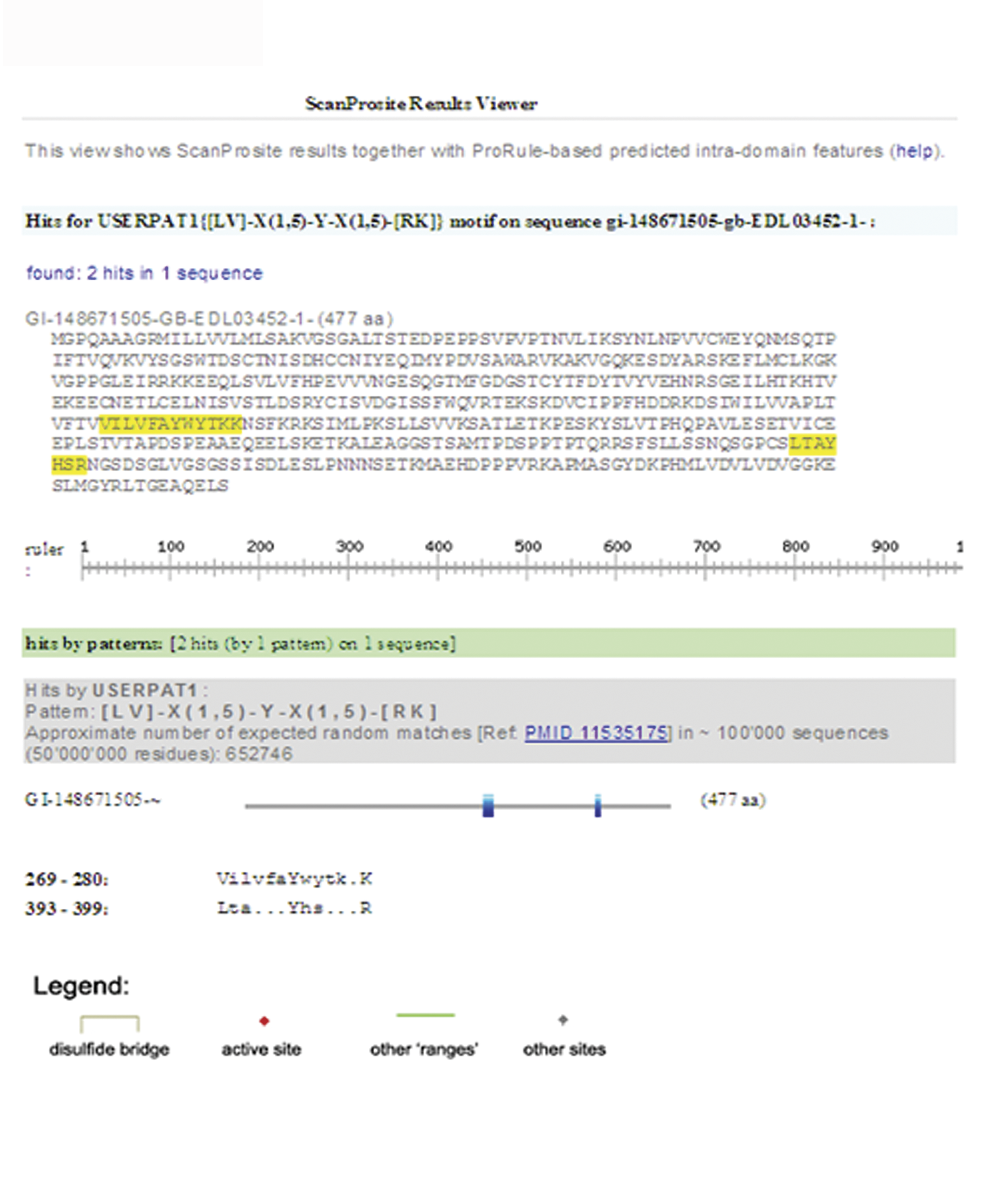

Supplement: Figure S4 — Scanprosite analysis of mouse IFNγR1 protein sequence (Accession: EDL03452.1 GI: 148671505) denoting the CRAC motifs. Two CRAC motifs in IFNγR1 protein sequence located within amino acid position −269 to −280 and −393 to −399 are highlighted in yellow. (TIF) [file ppat.1002229.s004.tif]

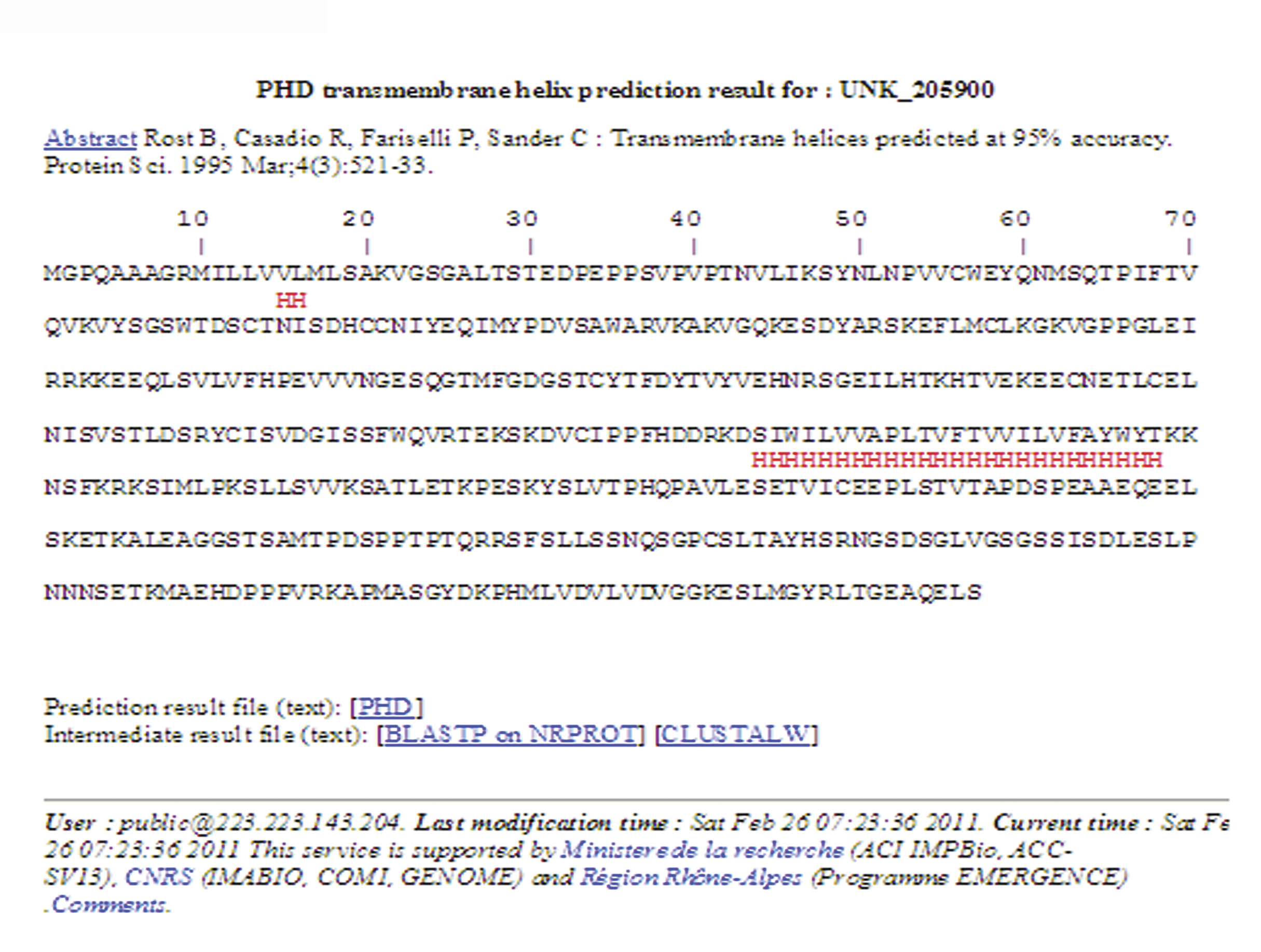

Supplement: Figure S5 — Transmembrane helix prediction result of mouse IFNγR1 protein sequence (Accession: EDL03452.1 GI: 148671505). The predicted α-helical portion of IFNγR1 protein sequence is denoted as letter ‘H’ in red. (TIF) [file ppat.1002229.s005.tif]

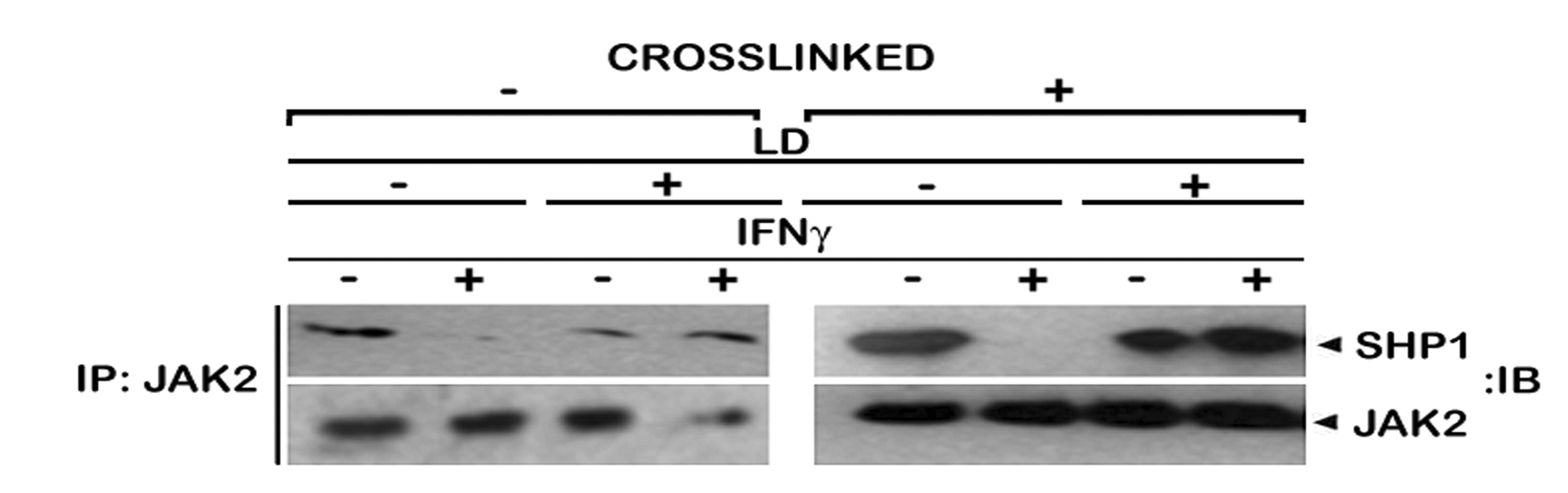

Supplement: Figure S6 — SHP-1 forms a constitutive complex with JAK2 in macrophages and the release of SHP-1 is IFNγ inducible. The MØs were infected with/without LD promastigotes for 12 hrs as described earlier, followed by treatment with 100 U/ml of rIFNγ for 3 minutes. Cells were then treated with/without cell permeable crosslinker of DSS, as per manufacture's protocol (Pierce). After cell lysis, protein extracts was immunoprecipitated with anti-JAK2. To verify the identity of JAK2 and SHP-1, immunoprecipitates were separated on polyacrylamide-SDS gel, and immunoblotting was performed with anti-JAK2 antibody or anti-SHP-1 antibody. (TIF) [file ppat.1002229.s006.tif]
