# Supplementary material for: Restoration of IFNγR Subunit Assembly, IFNγ Signaling and Parasite Clearance in Leishmania donovani Infected Macrophages: Role of Membrane Cholesterol
Source: PLoS Pathog. 2011 Sep 8;7(9):e1002229. doi: 10.1371/journal.ppat.1002229 (PMC3169561; doi:10.1371/journal.ppat.1002229)
Supplement: Table S1 — Identification of the cholesterol recognition / interacting amino acid consensus pattern. Alignment of amino acid sequences showing presence of CRAC motif conforming to -L/V-(X)1–5-Y-(X)1–5-R/K- in IFNγR1 proteins in different species. (DOC) [file ppat.1002229.s007.doc]

**Table S1: Identification of the cholesterol recognition/interacting amino acid consensus pattern in selected IFNγR1 protein**

| gi|148671505 *Mus musculus*(House mouse) | 269-**V**ilvfa**Y**wytk**K**-280 |
| --- | --- |
| gi|149039622 *Rattus novergicus*(Rat) | 269-**L**vcc**Y**ik**K**-276 |
| gi|632543 *Homo sapiens* (Human) | 258-**L**vficf**Y**ik**K**-267 |
| gi|197100085 *Pongo abelii* (Sumatran orangutan) | 263-**L**vficf**Y**ik**K**-272 |
| gi|295444941 *Sus scrofa* (pig) | 262-**L**ivac**Y**lir**K**-271 |
| gi|45385784 *Cervus elaphus* (red deer) | 61-**V**qvmn**Y**gdg**K**-70 |
| gi|296483981 *Bos taurus* (cattle) | 61-**V**qvmn**Y**edg**K**-70 |
| gi|118404146 *Xenopus (Silurana) tropicalis* (western clawed frog) | 184-**L**gcta**Y**ledll**K**-195 |
| gi|194332850 *Gallus gallus* ((chicken) | 255-**L**iltvc**Y**gckkl**R**-267 |
| gi|166406455 *Oncorhynchus mykiss* (rainbow trout) | 254-**V**wli**Y**s**K**-260 |

**Identification of the cholesterol recognition / interacting amino acid consensus pattern.** Alignment of amino acid sequences showing presence of CRAC motif conforming to

**-L/V-(X)_1–5_-Y-(X)_1–5_-R/K-** in IFNγR1 proteins in different species.
